# Preclinical comparison of (radio)lanthanides using mass spectrometry and nuclear imaging techniques: biodistribution of lanthanide-based tumor-targeting agents and lanthanides in ionic form

**DOI:** 10.1007/s00259-024-07018-9

**Published:** 2024-12-16

**Authors:** Rahel H. Wallimann, Avni Mehta, Ana Katrina Mapanao, Ulli Köster, Rainer Kneuer, Patrick Schindler, Nicholas P. van der Meulen, Roger Schibli, Cristina Müller

**Affiliations:** 1 Biomedical Research, Novartis, Basel 4056 Switzerland; 2 Department of Chemistry and Applied Biosciences, ETH Zurich, Zurich, 8093 Switzerland; 3 Center for Radiopharmaceutical Sciences, PSI Center for Life Sciences, Villigen-PSI, 5232 Switzerland; 4 Institut Laue-Langevin, Grenoble, 38042 France; 5 Laboratory of Radiochemistry, PSI Center for Nuclear Engineering and Sciences, Villigen-PSI, 5232 Switzerland

**Keywords:** Lanthanides, Lutetium, Terbium, Gadolinium, Europium, Multiplexed ICP-MS analysis, Dual-isotope SPECT imaging, Somatostatin receptor, Folate receptor, Prostate-specific membrane antigen

## Abstract

**Purpose:**

With the growing interest in exploring radiolanthanides for nuclear medicine applications, the question arises as to whether they are generally interchangeable without affecting a biomolecule’s pharmacokinetic properties. The goal of this study was to investigate similarities and differences of four (radio)lanthanides simultaneously applied as complexes of biomolecules or in ionic form.

**Methods:**

Inductively coupled plasma mass spectrometry (ICP-MS) was employed for the simultaneous detection of four lanthanides (Ln = lutetium, terbium, gadolinium and europium) in biological samples. In vitro tumor cell uptake and in vivo biodistribution studies were performed with Ln-DOTATATE, Ln-DOTA-LM3, Ln-PSMA-617 and Ln-OxFol-1. AR42J cells, PC-3 PIP cells and KB cells expressing the somatostatin receptor, the prostate-specific membrane antigen and the folate receptor, respectively, were used in vitro as well as to obtain the respective tumor mouse models for in vivo studies. The distribution of lanthanides in ionic form was investigated in immunocompetent mice. Dual-isotope SPECT/CT imaging studies were performed with mice administered with the radiolabeled biomolecules or chloride salts of lutetium-177 and terbium-161.

**Results:**

Similar in vitro cell uptake was observed for all four lanthanide complexes of each biomolecule into the respective tumor cell lines. AR42J tumor uptake of Ln-DOTATATE and Ln-DOTA-LM3 in mice showed similar values for all lanthanide complexes (3.8‒5.1% ID/g and 4.5‒5.0% ID/g; 1 h p.i., respectively). Accumulation of Ln-PSMA-617 in PC-3 PIP tumors (24–25% ID/g; 1 h p.i.) and of Ln-OxFol-1 in KB tumors (28–31% ID/g; 24 h p.i.) were also equal for the four lanthanide complexes of each biomolecule. After injection of lanthanide chloride salts (LnCl_3_; Ln = ^nat^Lu, ^nat^Tb, ^nat^Gd, ^nat^Eu), the liver uptake was different for each metal (~ 12% ID/g, ~ 22% ID/g, ~ 31% ID/g and ~ 37% ID/g; 24 h p.i., respectively) which could be ascribed to the radii of the respective lanthanide ions. In the bones, accumulation was considerably higher for lutetium than for other lanthanides (25 ± 5% ID/g vs. 14‒15% ID/g; 24 h p.i.). These data were confirmed visually by ^177^Lu/^161^Tb-based dual-isotope SPECT/CT images.

**Conclusions:**

The presented study confirmed similar properties of Ln-complexes, suggesting that lutetium-177 can be replaced by other radiolanthanides, most probably without affecting the tissue distribution profile of the resultant radiopharmaceuticals. On the other hand, the different radii of the lanthanide ions affected their uptake and resorption mechanisms in liver and bones when injected in uncomplexed form.

**Supplementary Information:**

The online version contains supplementary material available at 10.1007/s00259-024-07018-9.

## Introduction

In the field of nuclear oncology, there is growing interest in using radiolanthanides in combination with tumor target-specific biomolecules which makes them the preferred metals for the next-generation radiotheragnostics [1, 2]. The β¯-particle-emitting radiolanthanide, lutetium-177 (T_1/2_ = 6.65 d, E_β_^−^_average_ = 134 keV), is the most commonly used therapeutic radionuclide in clinics [3]. More recently, terbium-161, another radiolanthanide with similar decay characteristics (T_1/2_ = 6.95 d [4]; E_β_^−^_average_ = 154 keV) and γ-ray emission for SPECT imaging has gained considerable attention in the community [5, 6]. In addition to β¯-particles, terbium-161 also emits short-ranged electrons (conversion and Auger electrons), which are potentially beneficial for the treatment of micrometastases [7–9]. In general, lanthanides like lutetium and terbium exhibit similar chemical properties, thereby enabling their stable coordination with a DOTA chelator as demonstrated by similar logK values of 25.4 and 24.2 for Lu-DOTA and Tb-DOTA, respectively, along with other lanthanides including gadolinium and europium (Gd-DOTA: 24.6 and Eu-DOTA: 23.4) [10–12].

Several preclinical studies confirmed equal tissue distribution profiles of ^177^Lu- and ^161^Tb-based radiopharmaceuticals in mice [13–17]. Further exemplary studies focused on other radiolanthanides and showed the similarities of ^149^Pm- and ^166^Ho-labeled biotin or ^149^Pm- and ^153^Sm-labeled bombesin analogues to the ^177^Lu-based counterparts [18, 19]. Based on these findings, it can be assumed that any DOTA- or DOTAGA-functionalized biomolecule that is currently used with lutetium-177 could also be employed with terbium-161 and potentially other radiolanthanides without affecting its pharmacokinetic properties [19, 20]. In order to ultimately prove this hypothesis, a head-to-head comparison of several different radiolanthanides would be necessary. In principle, simultaneous measurement of radiolanthanides would, indeed, be feasible based on the characteristic γ-ray emission profile of each radiolanthanide using γ-spectrometry as previously reported [21]. However, a direct comparison of radiolanthanides other than lutetium-177 and terbium-161 is challenging due to their currently scarce availability and the fact that some radiolanthanides decay considerably faster than others [21, 22]. Comparison of lanthanide complexes of stable isotopes is more practical because of their commercial availability and the absence of decay-related factors, such as half-life and intensity of γ-ray emission. Additionally, naturally-occurring lanthanides can be detected at trace levels (ng/L) using inductively coupled plasma mass spectrometry (ICP-MS) [23–27]. Its multiplexing capability allows the quantification of more than one metal isotope at the same time, enabling the simultaneous detection of several lanthanides within the same sample [28–31].

The aim of this study was to employ ICP-MS for a direct comparison of four different lanthanides (Ln = lutetium, terbium, gadolinium or europium). Each biomolecule, namely DOTATATE, DOTA-LM3, PSMA-617 and OxFol-1 [32, 33], was labeled with the four lanthanides and applied as a cocktail for in vitro tumor cell uptake studies as well as for biodistribution studies in tumor-bearing mice. In addition, in vitro and in vivo studies were performed with the four lanthanides applied as chloride salts (LnCl_3_) or DTPA complexes (Ln-DTPA). Lutetium-177 and terbium-161, the two most relevant radiolanthanides in the context of medical applications, were investigated regarding their in vivo behavior using dual-isotope SPECT/CT imaging after application of the respective mixture of radiometal complexes or radiometal salts.

## Materials and methods

### Preparation of biomolecules labeled with naturally-occurring, stable lanthanide isotopes

DOTATATE [34], DOTA-LM3 [35], PSMA-617 [36] and OxFol-1 [32, 33, 37, 38] were labeled with the naturally-occurring isotopes of lutetium, terbium, gadolinium and europium (measured and herein indicated as lutetium-175, terbium-159, gadolinium-157 and europium-153, respectively) according to a previously published protocol [25]. ^nat^LuCl_3_, ^nat^GdCl_3_ and ^nat^EuCl_3_ were obtained from Sigma Aldrich Chemistry, Steinheim, Germany; ^nat^TbCl_3_ was obtained from ABCR GmbH, Karlsruhe, Germany. The identity of the products as well as quantitative and stoichiometric labeling of the tumor targeting agents was confirmed by mass determination using ultra performance liquid chromatography-mass spectrometry (UPLC-MS) (Supplementary Material). Solutions containing the Ln-labeled biomolecules were injected into an iron-free ultra performance liquid chromatography system, connected to a triple quadrupole ICP-MS instrument [25] to detect potential traces of uncoordinated metal ions. A chemical purity of ≥ 97% of all lanthanide-labeled tumor targeting agents was confirmed (Supplementary Material, Fig. S1-S3).

### ICP-MS analysis of cell and tissue samples

Cells and tissue samples were analyzed after microwave-assisted nitric acid digestion as previously reported [25]. In brief, cell and tissue samples were hydrolyzed using concentrated nitric acid (purified by redistillation, ≥ 99.999%, trace metal basis, Merck KGaA, Darmstadt, Germany). Holmium-165 (50 or 90 µL of a 0.1 mg/L solution in 2% (v/v) nitric acid) was added to each sample for the correction of potential matrix effects and the identification of volume losses during microwave digestion, which was performed at 150 °C for 10 min using a dedicated microwave system (Anton Paar Switzerland AG, Buchs, Switzerland). Samples were diluted using an ESI prepFAST precision M5x dilution system (Elemental Scientific, Inc. Ohama, NE, USA) and analyzed using a triple quadrupole ICP-MS instrument (iCAP TQ, Thermo Fisher, Reinach, Switzerland) as previously reported [25].

### In vitro studies

AR42J tumor cells, a somatostatin receptor (SSTR)-positive exocrine rat pancreatic cancer cell line (ECACC 93100618; Health Protection Agency Culture Collections, Salisbury, UK) [39], were cultured in Roswell Park Memorial Institute (RPMI) 1640 medium supplemented with glutamine, antibiotics and 20% fetal calf serum. Prostate-specific membrane antigen (PSMA)-positive PC-3 PIP and PSMA-negative PC-3 flu human prostate cancer cells were obtained from Prof. Dr. Martin Pomper (Johns Hopkins University School of Medicine, Baltimore, MD, USA). These cells were cultured in RPMI 1640 medium supplemented with glutamine, antibiotics, 10% fetal calf serum and puromycin. KB tumor cells, a folate receptor-positive cervical human cancer cell line (ACC-136; German Collection of Microorganisms and Cell cultures, DSMZ, GmbH, Germany) were cultured in RPMI 1640 cell culture medium with glutamine, antibiotics and 10% fetal calf serum, which was replaced at least one week prior to the in vitro studies or tumor cell inoculation by the folate-free analogous medium (FFRPMI, Cell Culture Technologies GmbH, Gravesano, Switzerland) with the respective supplements.

### In vitro tumor cell uptake studies

AR42J, PC-3 PIP and KB tumor cells were seeded in 6-well plates and left to grow overnight (Supplementary Material). The following day, a mixture of equal molar amounts of each Ln-DOTATATE (Ln = ^nat^Lu, ^nat^Tb, ^nat^Gd, ^nat^Eu) at a final concentration of 10 pmol/mL (2.5 pmol/mL per lanthanide complex) was added to each well containing adherent AR42J tumor cells using assay medium. The same procedure was performed with a mixture of the Ln-DOTA-LM3 complexes, the Ln-PSMA-617 complexes and the Ln-OxFol-1 complexes using AR42J, PC-3 PIP and KB tumor cells, respectively.

After a 1-h and 4-h incubation period, the tumor cells were rinsed 3 times with phosphate-buffered saline, pH 7.4 (PBS), to determine the total uptake of the biomolecules. The internalized fraction was assessed by rinsing the cells in addition with an acidic stripping buffer as previously reported [15, 37, 40]. Cells were detached using trypsin and transferred to microwave vials (MG5, 4 mL, Anton Paar Switzerland AG, Buchs, Switzerland) for microwave digestion and subsequent measurement using ICP-MS as described above (Supplementary Material).

A mixture of equal molar amounts of the lanthanide chloride salts (100 nM stock solutions of ^nat^LuCl_3_, ^nat^TbCl_3_, ^nat^GdCl_3_, and ^nat^EuCl_3_, diluted in saline with an adjusted pH value to 3.5‒4.0) was prepared to obtain a final concentration of 10 pmol/mL (2.5 pmol/mL per lanthanide salt) and tested with AR42J, PC-3 PIP and KB tumor cells according to the same protocol as described for the Ln-labeled biomolecules (Supplementary Material). Analogous in vitro cell uptake studies were also performed with lanthanide metal complexes of diethylenetriaminepentaacetic acid (DTPA, Sigma Aldrich Chemie, Steinheim, Germany) to obtain a final concentration of 10 pmol/mL (2.5 pmol/mL per lanthanide complex) (Supplementary Material).

Cell uptake studies were performed in triplicate in 3‒6 independent experiments and the results presented as the average ± standard deviation (SD). Data were normalized to the values measured for the ^175^Lu-labeled tumor targeting agent or [^175^Lu]LuCl_3_, which were set as 100% and zero was defined as 0%. Statistical significance of the normalized data was assessed for the comparison of the values obtained for terbium, gadolinium and europium analogues with those obtained for the lutetium counterparts by applying one-way ANOVA with the Dunnett’s post-test using GraphPad Prism software (version 8). A *p*-value of < 0.05 was considered statistically significant.

### In vivo studies

All animal experiments were performed in accordance with the guidelines of the Swiss Regulations for Animal Welfare following all applicable international, national, and/or institutional guidelines for the care and use of laboratory animals. Ethical approval was obtained from the Cantonal Committee of Animal Experimentation and licenses granted by the responsible cantonal authorities (N^o^ 75721 and N° 75668). Female CD1 nude mice, female athymic nude mice and female FVB mice were obtained at the age of 5–6 weeks from Charles River Laboratories (Sulzfeld, Germany). Experiments were performed after an acclimatization period of the mice of at least 7 days. Female CD1 nude mice were subcutaneously inoculated with AR42J or KB tumor cells (5 × 10^6^ cells in 100 µL PBS) on the right shoulder as previously reported [15, 37]. Female athymic nude mice were subcutaneously inoculated with PSMA-positive PC-3 PIP tumor cells (6 × 10^6^ cells in 100 µL Hank’s balanced salt solution (HBSS)) on the right shoulder and PSMA-negative PC-3 flu tumor cells (5 × 10^6^ cells in 100 µL HBSS) on the left shoulder [41]. Immunocompetent FVB mice were used without tumors. Biodistribution and SPECT/CT imaging studies were performed 2‒3 weeks after tumor cell inoculation. Mice with KB tumors were fed with a folate-deficient diet (ssniff Spezialdiäten GmbH, Soest, Germany) while all others received standard rodent chow.

### Biodistribution studies of lanthanide complexes and lanthanide salts

A mixture of Ln-labeled (Ln = ^nat^Lu, ^nat^Tb, ^nat^Gd and ^nat^Eu) tumor-targeting agents (2 nmol in total; 0.5 nmol of each lanthanide complex in 100 µL saline containing 0.05% bovine serum albumin (BSA)) was intravenously injected in corresponding tumor-bearing mice (*n* = 3‒4 mice per timepoint) [15, 37, 41]. The tissue distribution of the lanthanides was investigated in non-tumor-bearing immunocompetent FVB mice after intravenous injection of a mixture of all four LnCl_3_ in acidic solution (2 nmol in total, 0.5 nmol of each LnCl_3_, in 100 µL saline, pH 3.5 to 4.0). Mice were sacrificed after 1 h, 24 h or 96 h (*n* = 3 per timepoint). Ln-DTPA complexes were mixed as described for the lanthanide salts (2 nmol in total, 0.5 nmol for each Ln-DTPA complex) and diluted in 100 µL saline after adjustment of the pH value to 3.5 to 4.0. The FVB mice (*n* = 3) were sacrificed 1 h after intravenous injection of the Ln-DTPA complexes. Selected organs and tissues of mice were collected, weighed and processed for ICP-MS analysis as reported above (Supplementary Material) [25].

Biodistribution data were obtained from 3 to 4 mice per timepoint and the results presented as the average ± SD for each group. Data were normalized to the values measured for the Lu-labeled tumor-targeting agent, [^175^Lu]LuCl_3_ or [^175^Lu]Lu-DTPA, which were set as 100% and zero was defined as 0%. Statistical significance of the normalized data was assessed for organs and tissues with lanthanide accumulation > 0.5% ID/g, using one-way ANOVA with the Dunnett’s post-test in GraphPad Prism software (version 8). A *p*-value of < 0.05 was considered statistically significant.

### Radiolabeling of the biomolecules

Radiolabeling of DOTATATE, DOTA-LM3, PSMA-617 and OxFol-1 with lutetium-177 and terbium-161 was performed at a molar activity of 20 MBq/nmol under standard radiolabeling conditions at pH 4.5 as previously reported [13, 15, 17]. The radiolabeled biomolecules were obtained with a radiochemical purity of > 99% and used without further purification for in vivo dual-isotope SPECT/CT imaging studies. A 5-fold molar excess of DTPA relative to the tumor-targeting agent was added to prevent the presence of traces of unreacted or released radiolanthanide. L-ascorbic acid (3 mg) was added to the formulation of the radiolabeled biomolecules to protect them from radiolytic degradation (Supplementary Material).

### Dual-isotope SPECT/CT imaging

Dual-isotope SPECT studies with mice were performed as previously reported [15, 17]. Tumor-bearing nude mice (*n* = 2‒3 for each type of radioconjugate) were injected with 20 MBq (1 nmol) of a mixture of ^177^Lu- and ^161^Tb-labeled tumor-targeting agents (each 10 MBq, 0.5 nmol) diluted in saline containing 0.05% BSA. Non-tumor-bearing immunocompetent FVB mice (*n* = 2) were injected with 20 MBq of a mixture of [^177^Lu]LuCl_3_ (10 MBq, 13.8 pmol) and [^161^Tb]TbCl_3_ (10 MBq, 14.3 pmol) diluted with saline containing 0.05% BSA with a final pH value of 4.5. Additional mice (*n* = 2) were injected with colloids of lutetium-177 and terbium-161 obtained by diluting the chloride salts with PBS of pH 7.4 (Supplementary Material). SPECT/CT images were acquired 1 h, 4 h and 24 h after injection of the activity solutions using a dedicated small-animal SPECT/CT scanner (NanoSPECT/CT, Mediso Medical Imaging Systems, Budapest, Hungary) and the Nucline software (version 1.02, Mediso Ltd., Budapest, Hungary). Counts stemming from the γ-lines of lutetium-177 (112.9 keV ± 10% and 208.4 ± 10%) and the γ-lines and x-rays of terbium-161 (47.7 keV ± 10% and 74.6 keV ± 10%) were simultaneously registered using the respective energy windows for each radionuclide. Data were reconstructed based on either the γ-lines of lutetium-177 or the γ-lines and x-rays of terbium-161 to obtain separate distribution profiles for each radioconjugate as previously reported [15].

## Results

### Cell uptake of lanthanide-labeled tumor-targeting agents

**Fig. 1:**
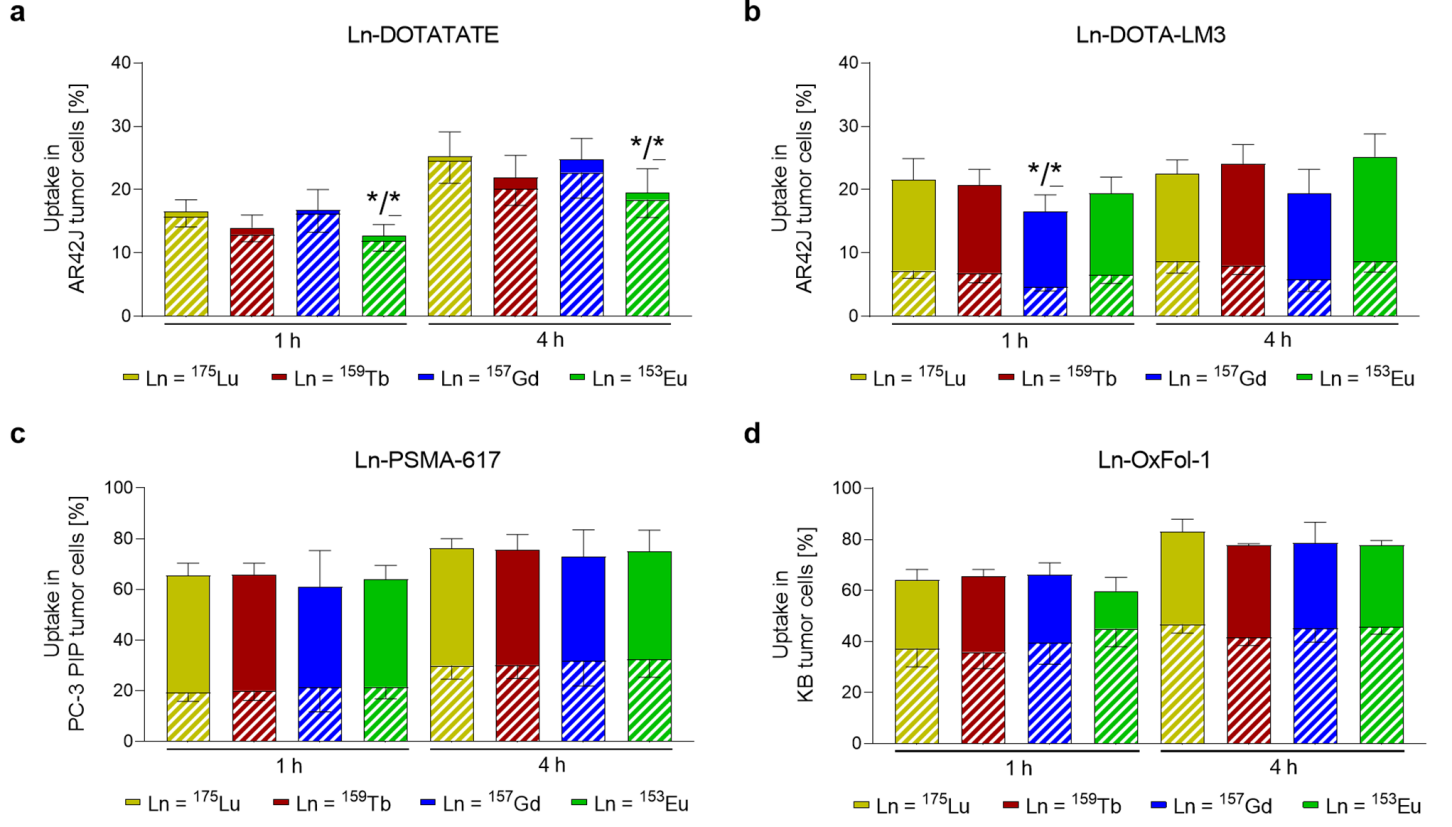
**a‒d** Total uptake (entire bars) and internalized fractions (hatched part of the bars) of Ln-labeled biomolecules. **a/b** Ln-DOTATATE and Ln-DOTA-LM3 in AR42J tumor cells; **c** Ln-PSMA-617 in PC-3 PIP tumor cells and **d** Ln-OxFol-1 in KB tumor cells. Data (average ± SD, *n* = 3–6) are presented as percentage of the added Ln-conjugate (set as 100%). Asterisks indicate uptake (*) and internalization (*) values that are significantly different from those measured for the respective ^175^Lu-labeled analogues (*p* < 0.05)

All four lanthanide complexes of each investigated biomolecule (Ln-DOTATATE, Ln-DOTA-LM3, Ln-PSMA-617 and Ln-OxFol-1; measured Ln = ^175^Lu, ^159^Tb, ^157^Gd and ^153^Eu) demonstrated similar cell uptake and internalization patterns (Fig. 1). Uptake of Ln-DOTATATE and Ln-DOTA-LM3 were within a comparable range of 15‒20% and 20‒25% of the total added amount of complex after a 1-h and 4-h incubation period, respectively, whereas effective internalization was only seen in the case of Ln-DOTATATE (~ 14% and ~ 21%, respectively) (Fig. 1a/b). Uptake of the Ln-PSMA-617 complexes reached 61‒66% (*p* > 0.05) and 73‒76% (*p* > 0.05) after a 1-h and 4-h incubation period, respectively, while the internalized fractions reached ~ 20% (*p* > 0.05) and ~ 30% (*p* > 0.05) after the same incubation periods (Fig. 1c). This also held true for Ln-OxFol-1 complexes, which showed 60‒66% (*p* > 0.05) and 78‒83% uptake (*p* > 0.05) after 1 h and 4 h incubation, respectively, with internalized fractions of ~ 40% (*p* > 0.05) and ~ 45% (*p* > 0.05) of total added complex (Fig. 1d).

### Cell uptake of lanthanide chloride salts and lanthanide DTPA complexes

Uptake of the lanthanide salts increased with time, however, it varied between the single lanthanide salts irrespective of the incubation period and tumor cell line (Supplementary Material, Fig. S4). Lanthanide DTPA complexes were neither bound nor internalized by tumor cells as demonstrated by the fact that only background levels of metals (< 20‒60 ppt) were detected in the cell samples (Supplementary Material).

### Biodistribution study of tumor-targeting agents labeled with naturally-occurring isotopes

**Fig. 2:**
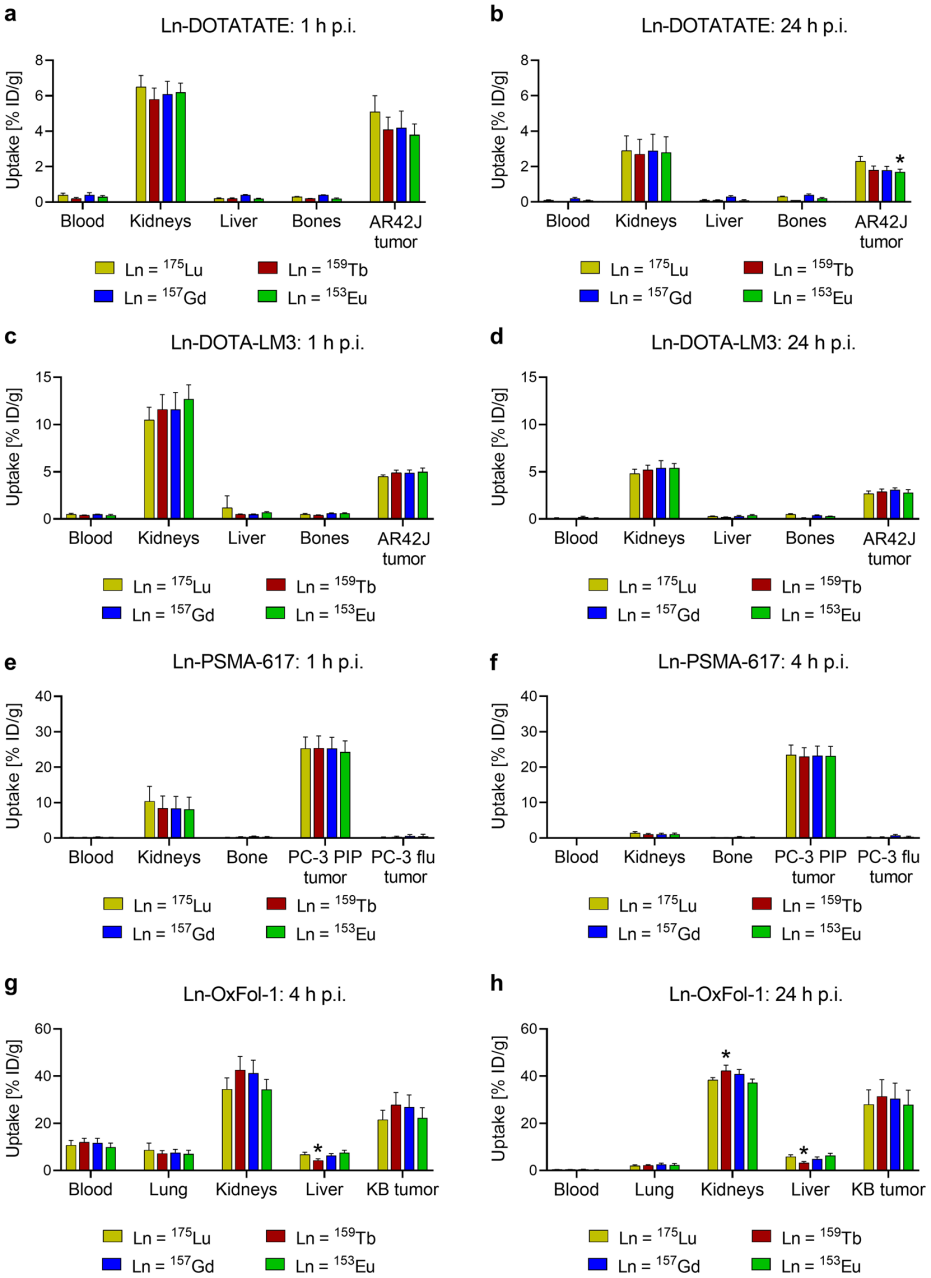
**a-h** Biodistribution data (average ± SD, *n* = 3–4) obtained at varied timepoints after injection of the mixture of lanthanide complexes (Ln = lutetium, terbium, gadolinium and europium; 0.5 nmol for each biomolecule) **a/b** DOTATATE and **c/d** DOTA-LM3 in AR42J tumor-bearing mice (1 h and 24 h p.i.), **e/f** PSMA-617 in PC-3 PIP and PC-3 flu tumor-bearing mice (1 h and 4 h p.i.) and **g/h** OxFol-1 in KB tumor-bearing mice (4 h and 24 h p.i.). Tissue uptake is expressed as percent injected dose per gram tissue [% ID/g]. Only organs and tissues relevant for the individual biomolecules are shown. Asterisks indicate uptake (*) values that are significantly different from those measured for the respective ^175^Lu-labeled biomolecules (*p* < 0.05)

Multiplexed ICP-MS analysis allowed the simultaneous investigation of biomolecules labeled with four naturally-occurring lanthanides in tumor-bearing animals (Supplementary Material, Fig. S5). In AR42J tumor-bearing mice, similar tissue distribution profiles were found for all Ln-DOTATATE complexes. Tumor accumulation was moderate (3.8‒5.1% ID/g at 1 h p.i. and 1.7‒2.3% ID/g at 24 h p.i.) and the uptake in the kidneys only slightly higher (5.8‒6.5% ID/g at 1 h p.i. and 2.7‒2.9% ID/g at 24 h p.i.). No significant differences were observed in the tissue distribution profiles of [^175^Lu]Lu-DOTATATE and [^159^Tb]Tb-DOTATATE (*p* > 0.05) and only minor fluctuations were observed between the data for [^153^Eu]Eu-DOTATATE and [^175^Lu]Lu-DOTATATE (Fig. 2a/b). Similar tissue distribution profiles were observed for all Ln-DOTA-LM3 complexes (*p* > 0.05) with slightly higher tumor uptake (4.5‒5.0% ID/g at 1 h p.i. and 2.7‒3.1% ID/g at 24 h p.i.) than observed for Ln-DOTATATE. Kidney uptake was also equal for all Ln-DOTA-LM3 complexes (11‒13% ID/g at 1 h p.i. and 4.8‒5.4% ID/g at 24 h p.i.) (Fig. 2c/d). Non-specific accumulation in off-target organs and tissues other than the kidneys was low and near the limit of quantification for all Ln-labeled somatostatin analogues (≤ 1% ID/g). Even though significant differences were observed (*p* < 0.05) in some of the off-target tissue, such as the spleen and the stomach, the absolute values were almost negligible (Supplementary Material, Table S1-S4).

The four Ln-PSMA-617 complexes demonstrated equal tissue distribution profiles, along with almost identical uptake in PC-3 PIP tumor xenografts (24‒25% ID/g at 1 h p.i. and 23% ID/g at 4 h p.i.; *p* > 0.05). Uptake in the PSMA-negative PC-3 flu tumor xenografts and normal tissues was low (≤ 1% ID/g) except for the kidneys (8.1‒10% ID/g at 1 h p.i. and 1.0‒1.4% ID/g at 4 h p.i.). As a result, differences in uptake were observed for single timepoints in the stomach and the spleen which were, however, small in absolute values (Fig. 2e/f, Supplementary Material, Table S5-S6).

Ln-OxFol-1 complexes also distributed similarly in KB tumor-bearing mice. Due to the albumin-binding properties of OxFol-1, blood retention was high (9.9‒12% ID/g at 4 h p.i.) and the accumulation in KB tumor xenografts increasing over time (22‒28% ID/g at 4 h p.i. and 28–31% ID/g at 24 h p.i.). Considerable uptake was seen in the lungs (7.0‒8.7% ID/g at 4 h p.i. and 2.0–2.6% ID/g at 24 h p.i.) and in the liver (4.3‒7.5% ID/g at 4 h p.i. and 3.3‒6.4% ID/g at 24 h p.i.). Significant differences (*p* < 0.05) were found for the liver uptake of [^175^Lu]Lu-OxFol-1 and [^159^Tb]Tb-OxFol-1 (6.8 ± 0.9% ID/g vs. 4.3 ± 0.6% ID/g at 4 h p.i. and 5.9 ± 0.7% ID/g vs. 3.3 ± 0.5% ID/g at 24 h p.i.) as well as for the retention in the kidneys (38 ± 1% ID/g vs. 42 ± 2% ID/g at 24 h p.i.) (Fig. 2g/h, Supplementary Material, Table S7-S8).

### Biodistribution study of lanthanide salts

**Fig. 3:**
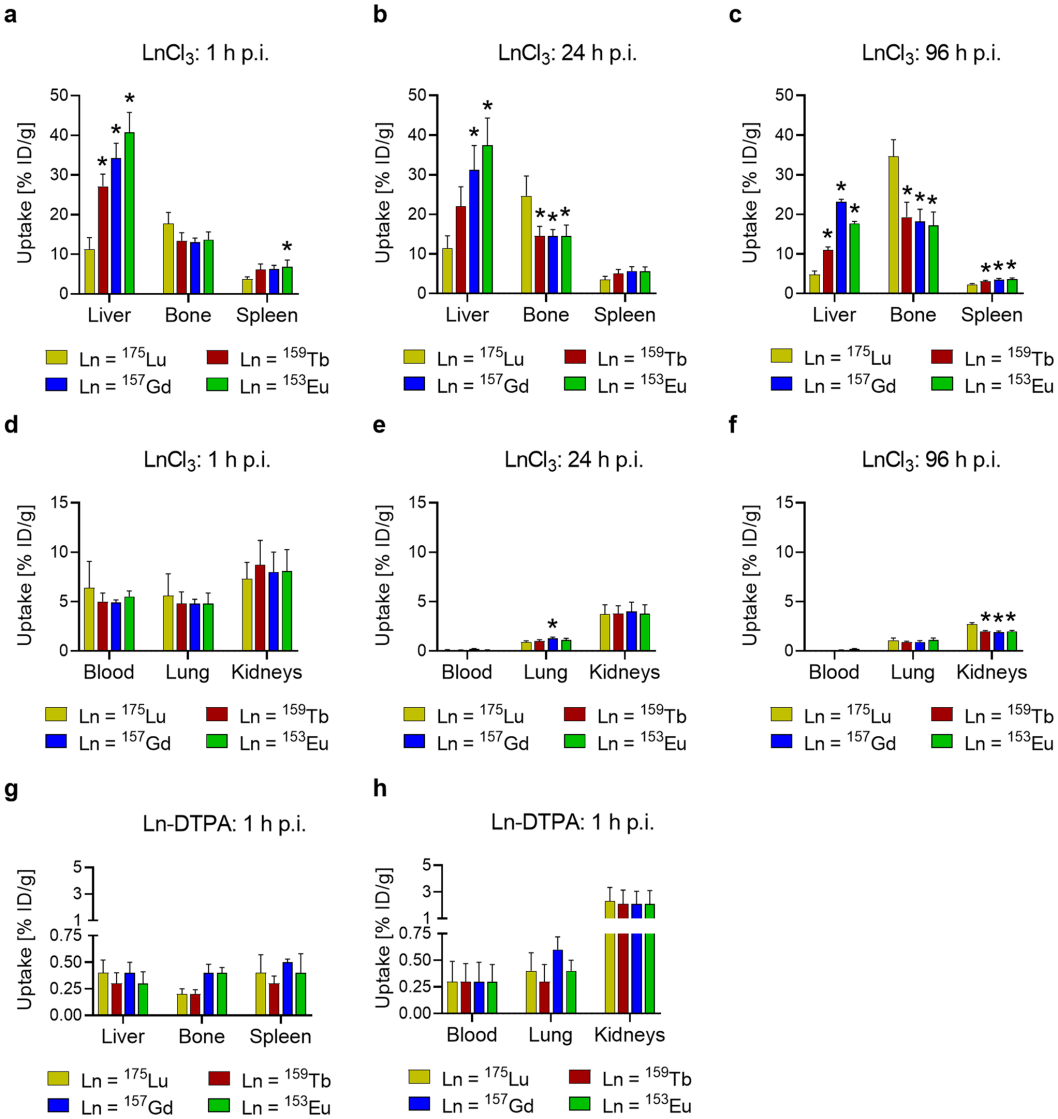
**a‒h** Biodistribution data (average ± SD, *n* = 3) obtained 1 h, 24 h, and 96 h after injection of chloride salts of naturally-occurring lutetium, terbium, gadolinium and europium (0.5 nmol each) in non-tumor-bearing mice. **a/b/c** Uptake in liver, bone and spleen; **d/e/f** uptake in blood, lungs and kidneys. Biodistribution data (average ± SD, *n* = 3) obtained 1 h after injection of DTPA-complexed naturally-occurring lutetium, terbium, gadolinium and europium (0.5 nmol each) in non-tumor-bearing mice. **g** Uptake in liver, bone and spleen; **h** uptake in blood, lung and kidneys. Tissue uptake is expressed as percent injected dose per gram tissue [% ID/g]. Asterisks indicate uptake (*) values that are significantly different from those measured for **a-f** [^175^Lu]LuCl_3_ or **g/h** [^175^Lu]Lu-DTPA (*p* < 0.05)

Application of naturally-occurring lanthanides in their chloride salt form (LnCl_3_) resulted in uneven distribution profiles. Hepatic accumulation was lowest for lutetium (11 ± 3% ID/g at 1 h p.i. and 12 ± 3% at 24 h p.i.) followed by terbium (27 ± 3% ID/g at 1 h p.i. and 22 ± 5% at 24 h p.i.), gadolinium (34 ± 4% ID/g at 1 h p.i. and 31 ± 6% at 24 h p.i.) and europium (41 ± 5% ID/g at 1 h p.i. and 37 ± 7% at 24 h p.i.) (*p* < 0.05). The same trend was visible in the spleen, even though the uptake was much lower. Bone accumulation of the lanthanides was increasing over time. At the 96 h p.i. timepoint, the accumulation of lutetium in the bones was about ~ 2-fold higher (35 ± 4% ID/g) than for terbium (19 ± 4% ID/g), gadolinium (17 ± 3% ID/g) and europium (18 ± 3% ID/g) (*p* < 0.05) (Fig. 3a-c). Also in the kidneys, higher retention was observed after injection of lutetium (2.7 ± 0.2% ID/g at 96 h p.i.) than after application of the other lanthanides (1.9‒2.0% ID/g at 96 h p.i.; *p* < 0.05). All lanthanides demonstrated equal retention in the blood (4.9‒6.4% ID/g at 1 h p.i.) and the lungs (4.8‒5.6% ID/g at 1 h p.i.) at the early time points and were effectively cleared over time (Fig. 3d-f, Supplementary Material, Tables S9-S11).

Ln-DTPA complexes were rapidly cleared from the blood circulation and barely detectable in most organs and tissues even as early as 1 h after injection (< 1% ID/g at 1 h p.i.). Only in the kidneys through which the Ln-DTPA complexes were excreted, the lanthanide complexes were still detectable at low concentrations (2.1‒2.3% ID/g at 1 h p.i.; *p* > 0.05), (Fig. 3g/h, Supplementary Material, Table S12).

### Dual-isotope SPECT/CT imaging

**Fig. 4:**
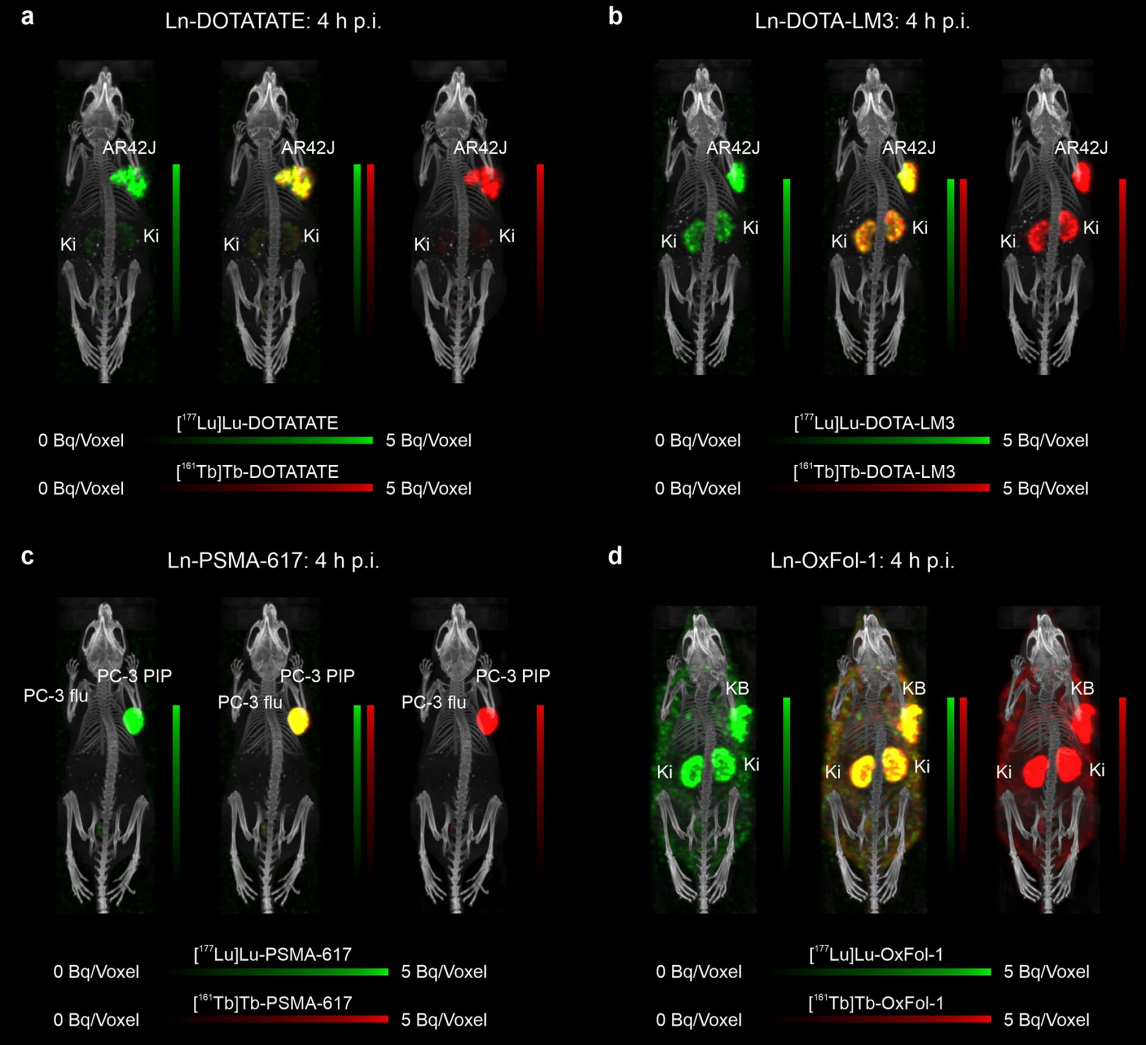
**a‒d** Dual-isotope SPECT/CT images of tumor-bearing mice injected with a mixture of ^177^Lu-labeled (10 MBq, 0.5 nmol) and ^161^Tb-labeled (10 MBq, 0.5 nmol) biomolecules at 4 h p.i. **a** AR42J tumor-bearing mouse injected with Ln-DOTATATE; **b** AR42J tumor-bearing mouse injected with Ln-DOTA-LM3; **c** PC-3 PIP/flu tumor-bearing mouse injected with Ln-PSMA-617; **d** KB tumor-bearing mouse injected with Ln-OxFol-1. (AR42J, SSTR-positive tumor xenograft; PC-3 PIP, PSMA-positive tumor xenograft; PC-3 flu, PSMA-negative tumor xenograft; KB, folate receptor-positive tumor xenograft; Ki, kidney)

**Fig. 5:**
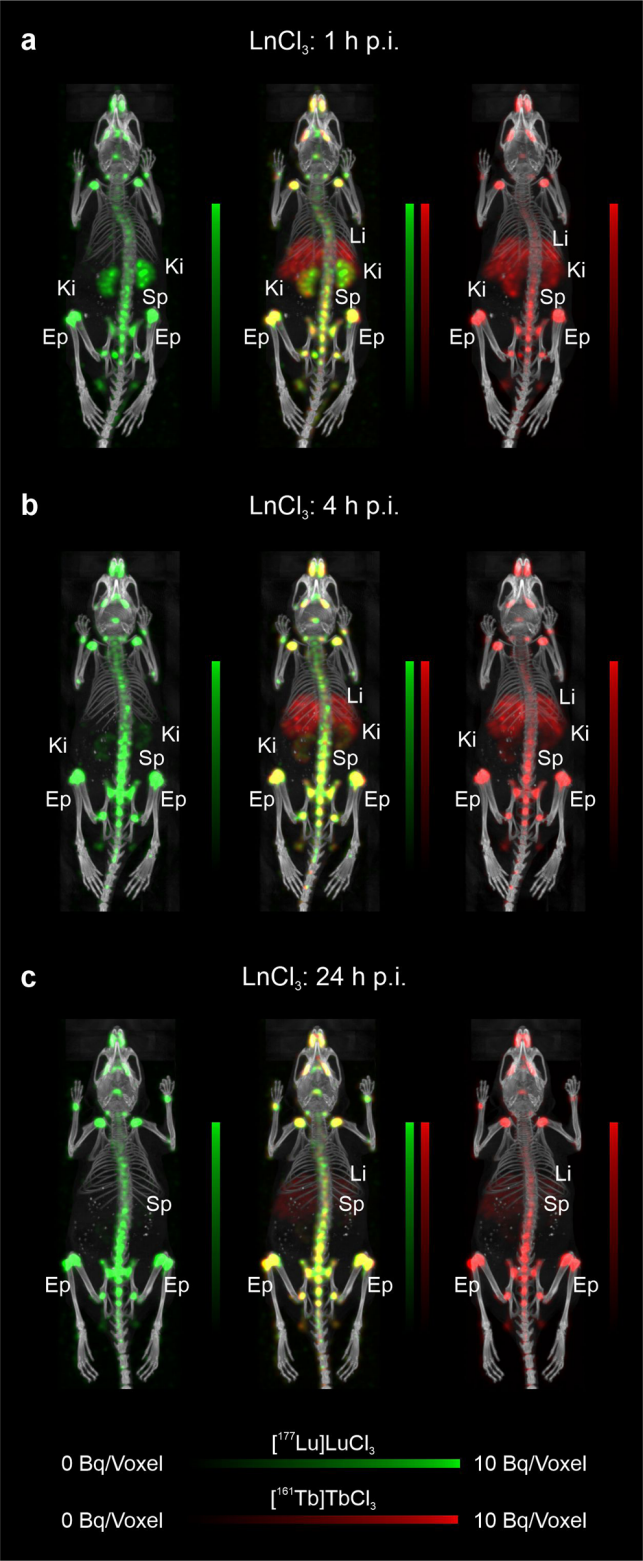
**a**‒**c** Dual-isotope SPECT/CT images of an FVB mouse injected with a mixture of [^177^Lu]LuCl_3_ (10 MBq, 13.8 pmol) and [^161^Tb]TbCl_3_ (10 MBq, 14.3 pmol) in acidic formulation. **a** Scan acquired at 1 h p.i.; **b** scan acquired at 4 h p.i. and **c** scan acquired at 24 h p.i. (Ki, kidney; Li, liver; Sp, spine; Ep, epiphysis (only indicated in knee joint))

Representative dual-isotope SPECT/CT images of tumor-bearing mice showed nearly identical tissue distribution profiles of the co-administered ^177^Lu- and ^161^Tb-labeled biomolecules (Fig. 4, Supplementary Material, Fig. S6-S9). This was not the case upon injection of the radiolanthanide salts, which resulted in an intense uptake and prolonged retention of lutetium in the bones, in particular in the epiphysis. While terbium accumulated less in the bone, it resulted in higher uptake and retention in the liver (Fig. 5, Supplementary Material, Fig. S10). These results are in line with the biodistribution studies that were performed with the respective metal-labeled biomolecules and metal salts of the stable lanthanide isotopes. On the other hand, if the radiolanthanides were administered as colloids in a pH-neutral formulation, an equal accumulation pattern in liver and spleen was seen for lutetium-177 and terbium-161 (Supplementary Material, Fig. S11).

## Discussion

This study revealed similar cellular uptake of each Ln-labeled biomolecule irrespective of whether lutetium or any other lanthanide was used. In vivo biodistribution data further confirmed the hypothesis that the tissue distribution profile of a biomolecule was comparable for all investigated lanthanides. Significant differences among the four Ln-labeled versions of each biomolecules were observed in a few cases of non-targeted tissues, in which the uptake was generally low and the measurement of the respective lanthanide close to the detection limit. Such variabilities could potentially be resolved by using larger cohorts of animals in these experiments.

In line with previously performed biodistribution studies using lanthanides applied in salt form at acidic pH [42–46], our study demonstrated preferential accumulation of the lanthanides in the liver and bones of mice. Liver uptake of the herein employed lanthanide chloride salts (Lu: 12% ID/g, Tb: 22% ID/g, Gd: 31% ID/g and Eu: 37% ID/g at 24 h p.i., respectively) correlated positively with their ionic radii (Lu: 0.861 Å, Tb: 0.923 Å, Gd: 0.938 Å and Eu: 0.947 Å [47]) which was in agreement with previously reported data [21]. It is, thus, likely that the hepatic uptake was related to the binding of lanthanide ions to Ca-dependent carriers because of the similarity in ionic radii between lanthanide and calcium ions [47–49]. If the lanthanides were, however, applied in colloid form at neutral pH, they were previously found to accumulate in the liver and spleen as a result of the activity of phagocytic Kupffer cells and splenic macrophages [42, 45].

Furthermore, previous investigations showed that as the ionic radius of the lanthanides decreases and the charge density increases, they exhibit a more favorable exchange with calcium ions in the hydroxyapatite binding sites [50–52]. This may explain the observation of the considerably higher bone accumulation of lutetium compared to the other investigated lanthanides. The observation of liver and bone accumulation of lanthanide ions underlines the relevance of preventing the presence of uncoordinated radiolanthanides in formulations of radiopharmaceuticals, which can be guaranteed by chelation of potential traces of non-reacted or released radiolanthanides [21]. Therefore, the addition of DTPA to radiopharmaceutical formulations has been suggested to capture traces of uncoordinated lutetium-177 [42, 50, 53]. Indeed, our data demonstrated the fast renal excretion of DTPA lanthanide complexes as previously reported for [^177^Lu]Lu-DTPA [53].

The underlying idea of using other lanthanides as a surrogate for lutetium ‒ based on the assumption that this would not change the properties of the metal complex ‒ has previously been demonstrated by Holtzapfel et al. who used europium instead of lutetium to evaluate PSMA-targeting DOTA conjugates by means of ICP-MS [24]. This suggests the feasibility of using the ICP-MS methodology for the simultaneous screening process of multiple drug candidates labeled with different lanthanides.

Cassette dosing experiments using multiplexed ICP-MS for the detection of multiple elements in biological material could accelerate the drug screening process and eliminate inter-individual differences among the test animals [54]. Importantly, this methodology could significantly reduce the number of test animals, and hence, contribute-AMh substantially to the 3R principle (“Replace, Reduce, Refine”). Despite the high sensitivity of ICP-MS, this methodology may, however, be limited by the relatively large quantities of metal conjugates that are necessary for accurate quantification. This may comprise a risk of receptor saturation in organs with moderate to low receptor expression levels [25]. Moreover, the simultaneous investigation of multiple metal complexes may result in unwanted interactions and affect their distribution profiles [54, 55].

In radiopharmaceutical sciences, the methodology of dual-isotope SPECT imaging is a similar concept which is based on the measurement of γ-ray emission of two different radionuclides (e.g. lutetium-177 and terbium-161). This method can allow the simultaneous investigation of biomolecules labeled with two different radionuclides for longitudinal studies [15, 17]. The present study reinforces the utility of this imaging technology by visualizing the equal in vivo distribution of a mixture and ^177^Lu- or ^161^Tb-labeled biomolecules. SPECT images also confirmed the biodistribution data obtained by ICP-MS measurements, demonstrating the increased liver accumulation of terbium-161 but higher bone uptake for lutetium-177 after application of these radiolanthanides in chloride form. When applied as colloids, the accumulation pattern was, however, similar for both radiolanthanides with uptake seen almost exclusively in liver and spleen, in agreement with previous findings of others who applied the lanthanides as colloids at neutral pH [42, 45]. In line with previous studies conducted in rats [34] and dogs [56], our imaging data showed increased localization of the radiolanthanides in the epiphyseal plates as a result of the extensive metal ion resorption. Therefore, apart from revealing the whole-body biodistribution of the radioligands in question over time, dual-isotope SPECT/CT imaging has the advantage of providing information on sub-organ distribution.

## Conclusion

Multiplexed ICP-MS analysis allowed simultaneous detection and, hence, direct comparison of co-injected tumor-targeting agents labeled with different lanthanides. While lanthanides in ionic form may have distinct biodistribution patterns due to varying radii and charge densities, only marginal variations arise in their chelator-complexed form. It is, thus, likely that radiolanthanides beyond lutetium-177 and terbium-161 could be interchanged without affecting the biological behavior of the respective radiopharmaceuticals. The comparable behavior of lutetium, terbium, gadolinium and europium in their chelated form opens the possibility of screening drug candidates simultaneously in view of their potential as radiopharmaceuticals.

## Electronic supplementary material

Below is the link to the electronic supplementary material.

Supplementary Material 1

## Data Availability

All preclinical data analyzed in this study are included in this published article and in the Supplementary Material. Additional information or more detailed data are available from the corresponding author on reasonable request.
