# Supplementary material for: Preclinical comparison of (radio)lanthanides using mass spectrometry and nuclear imaging techniques: biodistribution of lanthanide-based tumor-targeting agents and lanthanides in ionic form
Source: Eur J Nucl Med Mol Imaging. 2024 Dec 16;52(4):1370–82. doi: 10.1007/s00259-024-07018-9 (PMC11839852; doi:10.1007/s00259-024-07018-9)
Supplement: Supplementary file 1 — Supplementary Material 1 [file 259_2024_7018_MOESM1_ESM.docx]

**Supplementary Material**

**Preclinical comparison of (radio)lanthanides using mass spectrometry and nuclear imaging techniques: biodistribution of lanthanide-based tumor-targeting agents and lanthanides in ionic form**

Rahel H. Wallimann^1,2†^, Avni Mehta^3†^, Ana Katrina Mapanao^3^, Ulli Köster^4^, Rainer Kneuer^1^, Patrick Schindler^1^, Nicholas P. van der Meulen^3,5^, Roger Schibli^2,3^, Cristina Müller^2,3^*

^1^ Biomedical Research, Novartis, 4056 Basel, Switzerland

^2^ Department of Chemistry and Applied Biosciences, ETH Zurich, 8093 Zurich, Switzerland

^3^ Center for Radiopharmaceutical Sciences, PSI Center for Life Sciences, 5232 Villigen-PSI, Switzerland

^4^ Institut Laue-Langevin, 38042 Grenoble, France

^5^ Laboratory of Radiochemistry, PSI Center for Nuclear Engineering and Sciences, 5232 Villigen-PSI, Switzerland

^†^ equally contributed

**Address of the corresponding author**

Prof. Dr. Cristina Müller

Center for Radiopharmaceutical Sciences

PSI Center for Life Sciences

5232 Villigen-PSI, Switzerland

E-mail: [cristina.mueller@psi.ch](mailto:cristina.mueller@psi.ch)

Phone: +41 56 310 44 54

**1. Labeling of the biomolecules with the naturally-occurring lanthanides**

***Purpose***: The biomolecules DOTATATE, DOTA-LM3, PSMA-617, and OxFol-1 were labeled with the naturally-occurring isotopes of lutetium, terbium, gadolinium and europium.

***Methods***: *Somatostatin analogues:* A stock solution of DOTATATE (ABX advanced biomedical compounds 9770, Radeberg, Germany) was prepared by dissolving the peptide in Milli-Q water containing 5% (v/v) dimethylsulfoxide to obtain a final concentration of 1 mM. A stock solution of DOTA-LM3 (Silicon Valley Menlo Park, CA, USA) was prepared by dissolving the peptide in Milli-Q water to obtain a final concentration of 1 mM. These stock solutions were diluted in 50 mM NaOAc buffer, pH 5.5 to obtain a concentration of 0.1 mM. The peptides were subsequently labeled with ^nat^LuCl_3_ (Sigma Aldrich Chemistry, Steinheim, Germany), ^nat^TbCl_3_ (ABCR GmbH, Karlsruhe, Germany), ^nat^GdCl_3_ (Sigma Aldrich Chemistry, Steinheim, Germany) and ^nat^EuCl_3_ (Sigma Aldrich Chemistry, Steinheim, Germany) using one molar equivalent of the metal salt dissolved in 0.05 M hydrochloric acid. The reaction mixtures were incubated at 95 °C for 10 min to obtain quantitative ^nat^Lu-, ^nat^Tb-, ^nat^Gd- and ^nat^Eu-labeling of DOTATATE and DOTA-LM3.

*PSMA ligand*: A stock solution of PSMA-617 (synthesized by the group at ETH Zurich) was prepared by dissolving the ligand in Milli-Q water to obtain a final concentration of 1 mM. This stock solution was diluted in 50 mM NaOAc buffer, pH 5.5, to obtain a concentration of 0.1 mM. The labeling with the respective lanthanide salts was performed as described for the somatostatin analogues.

*Folate conjugate*: A stock solution of OxFol-1 (synthesized by the group at ETH Zurich [1, 2]) was prepared by dissolving the folate conjugate in Milli-Q water containing 5% (v/v) 0.5 M NaOAc to obtain a final concentration of 1 mM. This stock solution was diluted in 50 mM NaOAc buffer, pH 5.5, to obtain a concentration of 0.1 mM. The labeling with the respective lanthanide salts was performed as described for the somatostatin analogues.

The identity of the products was confirmed using ultra performance liquid chromatography-mass spectrometry (UPLC-MS). Quality control of the products was performed as previously reported [3]. In brief, an aliquot of the respective product was diluted in diethylenetriamine pentaacetic acid (DTPA; 50 µM, aq.) for subsequent analysis using an iron-free ultra-performance liquid chromatography (UPLC) system coupled to an ICP-MS (iCAP TQ, Thermo Fisher, Reinach, Switzerland). The UPLC system was equipped with a quaternary pump, a split sampler, a variable wavelength detector (Vanquish flex, Thermo Fisher Scientific, Reinach, Switzerland), and a C-18 reversed phase column (Halo Peptide, 160 Å, ES-C18, 2.7 µm, 1.0 x 75 mm, Waters, Wilmington, DE, USA). A linear gradient of Milli-Q water containing 0.1% trifluoroacetic acid (98-20%) and methanol (2-80%) was used over 10 min at a flow rate of 0.1 mL/min. The ICP-MS was equipped with a perfluoroalkoxy liquid chromatography integrated capillary valve nebulizer (Elemental Scientific, Inc. Ohama, NE, USA) and a cyclonic quartz spray chamber (Thermo Fisher Scientific, Reinach, Switzerland) with the spray chamber temperature set to 2.7 °C. When the ICP-MS was used in combination with reversed-phase chromatography, a platinum skimmer cone and a platinum sampler cone (Thermo Fisher Scientific, Reinach, Switzerland) were used. A solution containing thulium-169 was constantly infused into the ICP-MS with a flow rate of 0.05 mL/min to monitor the performance of the ICP-MS. Measurements by ICP-MS were performed in single quadrupole mode applying helium as non-reactive gas for focusing and detection of lutetium-175 and thulium-169 ions. For the detection of terbium-159 and gadolinium-157, the measurements were performed in triple quadrupole mode applying oxygen as a reactive gas. For the detection of europium-153, the measurements were performed in single quadrupole mode while applying the kinetic energy discrimination mode.

***Results***: *Somatostatin analogues:* Stoichiometric labeling of DOTATATE and DOTA-LM3 was achieved with the naturally-occurring isotopes of lutetium, terbium, gadolinium and europium. The masses of ^175^Lu, ^159^Tb-, ^157^Gd-, and ^153^Eu-labeled DOTATATE detected by UPLC-MS were m/z = 1608.5 [M+H]^+^, m/z = 1592.5 [M+H]^+^, m/z = 1590.5 [M+H]^+^, and m/z = 1586.5 [M+H]^+^, respectively. The detected masses of ^175^Lu-, ^159^Tb-, ^157^Gd-, and ^153^Eu-labeled DOTA-LM3 were m/z = 1722.7 [M+H]^+^, m/z = 1706.6 [M+H]^+^, m/z = 1704.7 [M+H]^+^, and m/z = 1700.6 [M+H]^+^, respectively. Quality control of the final products revealed high chemical purity of ≥99% for all lanthanide complexes (Fig. S1).


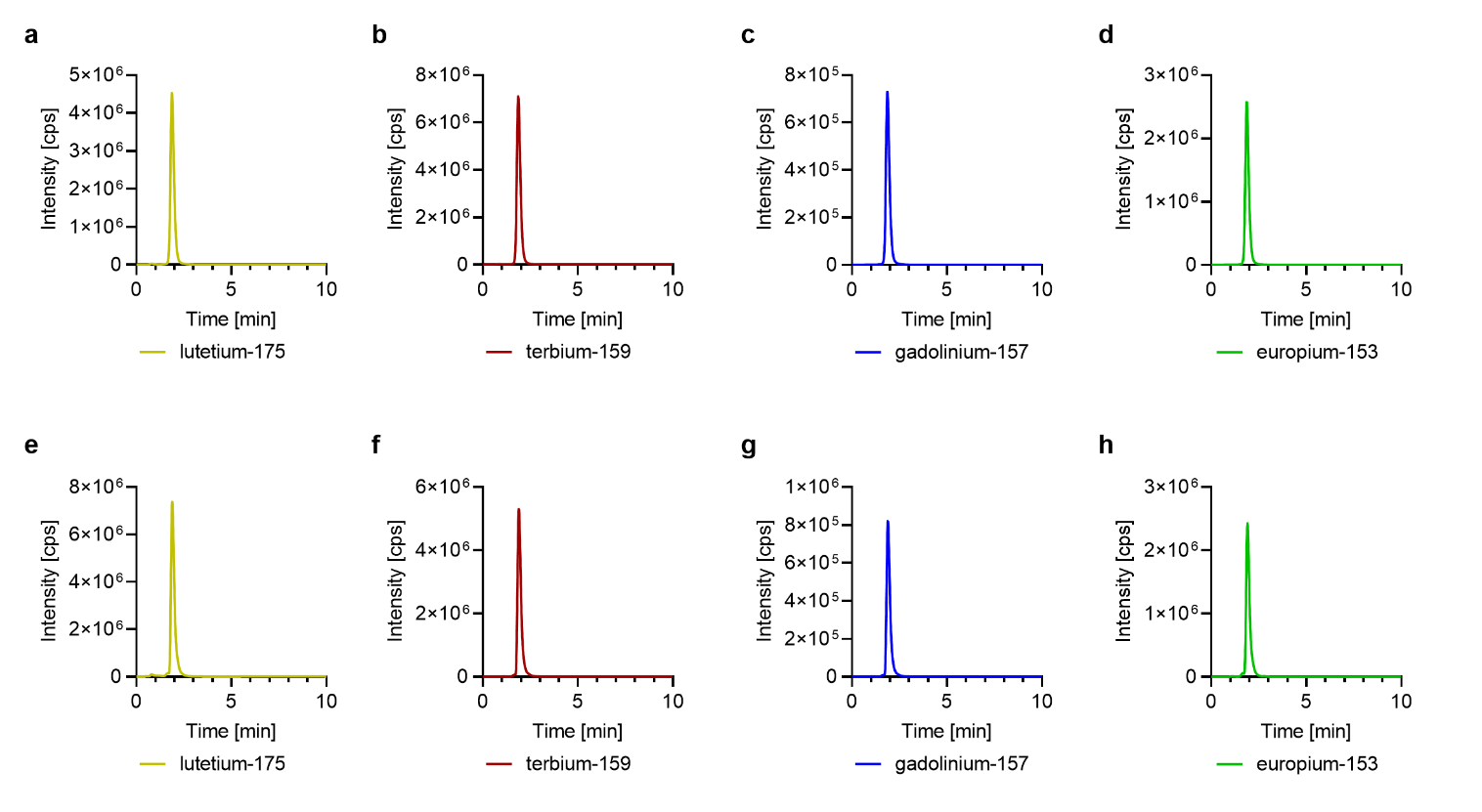


**Fig. S1** **a‒h** Representative chromatograms obtained by UPLC-ICP-MS analysis of lanthanide complexes of somatostatin analogues. **a** [^175^Lu]Lu-DOTATATE (t_R_ = 1.9 min), **b** [^159^Tb]Tb-DOTATATE (t_R_ = 1.9 min), **c** [^157^Gd]Gd-DOTATATE (t_R_ = 1.9 min), **d** [^153^Eu]Eu-DOTATATE (t_R_ = 1.9 min), **e** [^175^Lu]Lu-DOTA-LM3 (t_R_ = 1.9 min), **f** [^159^Tb]Tb-DOTA-LM3 (t_R_ = 1.9 min), **g** [^157^Gd]Gd-DOTA-LM3 (t_R_ = 1.9 min) and **h** [^153^Eu]Eu-DOTA-LM3 (t_R_ = 1.9 min). Uncoordinated metal salts complexed by DTPA would elute at a retention time of t_R_ = 0.8‒1.3 min

*PSMA ligand:* Stoichiometric labeling of PSMA-617 was achieved with the naturally-occurring isotopes of lutetium, terbium, gadolinium and europium. The detected masses of ^175^Lu-, ^159^Tb-, ^157^Gd-, and ^153^Eu-labeled PSMA-617 were m/z = 1215.4 [M+H]^+^, m/z = 1199.4 [M+H]^+^, m/z = 1198.4 [M+H]^+^, and m/z = 1193.4 [M+H]^+^, respectively. Quality control of the final products revealed high chemical purity of ≥99% for all lanthanide complexes (Fig. S2).


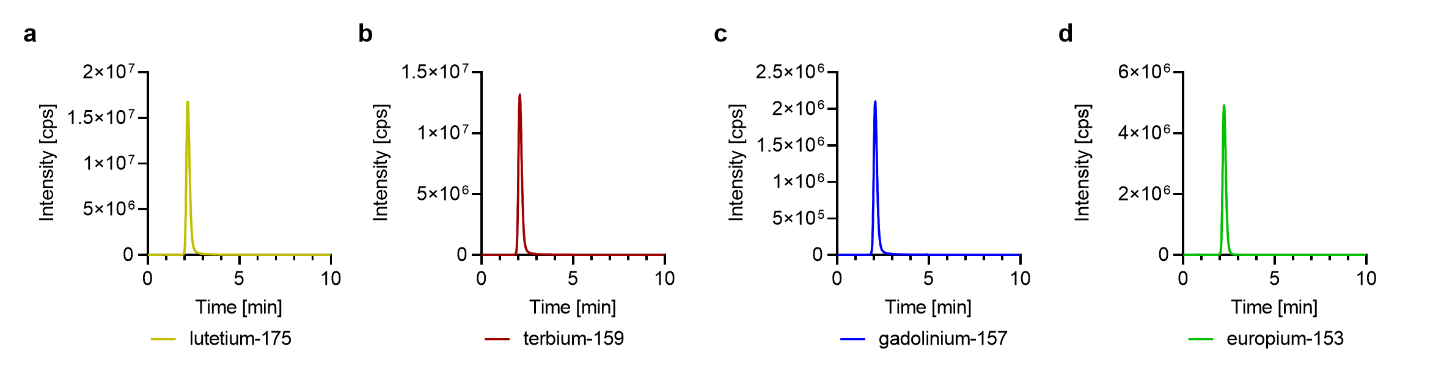


**Fig. S2** **a‒d** Representative chromatograms obtained by UPLC-ICP-MS analysis of lanthanide complexes of PSMA-617. **a** [^175^Lu]Lu-PSMA-617 (t_R_ = 2.2 min), **b** [^159^Tb]Tb-PSMA-617 (t_R_ = 2.1 min), **c** [^157^Gd]Gd-PSMA-617 (t_R_ = 2.1 min), and **d** [^153^Eu]Eu-PSMA-617 (t_R_ = 2.2 min). Uncoordinated metal salts complexed by DTPA would elute at a retention time of t_R_ = 0.8‒1.3 min

*Folate conjugate:* Stoichiometric labeling of OxFol-1 was achieved with the naturally-occurring isotopes of lutetium, terbium, gadolinium and europium was achieved. The masses of ^175^Lu-, ^159^Tb-, ^157^Gd-, and ^153^Eu-labeled OxFol-1 were m/z = 1528.5 [M+H]^+^, m/z = 1513.4 [M+H]^+^, m/z = 1512.4 [M+H]^+^, and m/z = 1507.4 [M+H]^+^, respectively. Quality control of the final products revealed high chemical purity of ≥97% for all lanthanide complexes (Fig. S3).


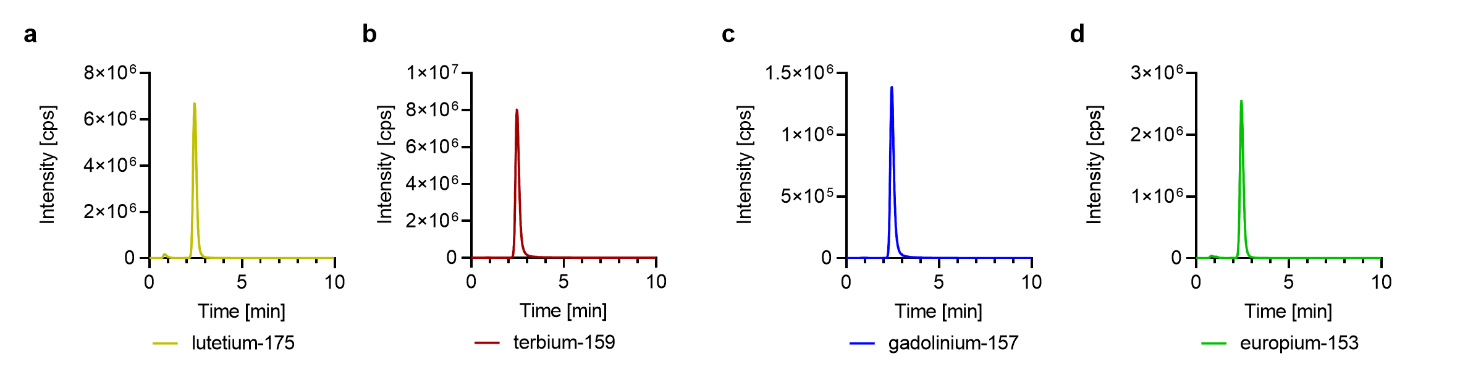


**Fig. S3** **a‒d** Representative chromatograms obtained by UPLC-ICP-MS analysis of lanthanide complexes of OxFol-1. **a** [^175^Lu]Lu-OxFol-1 (t_R_ = 2.4 min), **b** [^159^Tb]Tb-OxFol-1 (t_R_ = 2.5 min), **c** [^157^Gd]Gd-OxFol-1 (t_R_ = 2.5 min), and **d** [^153^Eu]Eu-OxFol-1 (t_R_ = 2.4 min). Uncoordinated metal salts complexed by DTPA would elute at a retention time of t_R_ = 0.8‒1.3 min

**2. In vitro cell uptake studies of Ln-labeled tumor-targeting agents**

***Purpose***: In vitro cell uptake and internalization experiments were performed with Ln-labeled DOTATATE and DOTA-LM3, as well as PSMA-617 and OxFol-1.

***Methods***: The day before the experiment, somatostatin receptor (SSTR)-positive AR42J tumor cells, prostate-specific membrane antigen (PSMA)-transfected PC-3 PIP tumor cells and folate receptor-positive KB tumor cells were seeded in 6-well plates at 3.5 × 10^6^, 1 × 10^6^ and 2.5 × 10^6^ cells per well, in 4 mL of the corresponding cell culturing medium. In the case of AR42J tumor cells, poly-lysine coated well plates were used. The tumor cells were allowed to adhere to the well plates overnight at 37 °C and 5% CO_2_. The next day, the medium was removed and the tumor cells were rinsed with phosphate-buffered saline, pH 7.4 (PBS), before addition of the respective assay medium (975 µL). Assay medium was defined separately for each cell line: In the case of AR42J tumor cells, assay medium was defined as RPMI medium supplemented with 1% fetal calf serum (FCS). In the case of PC-3 PIP tumor cells, assay medium referred to RPMI medium without additives and in the case of KB cells, assay medium was defined as folate-free RPMI (FFRPMI) medium without additives.

The respective Ln-labeled biomolecules were mixed in assay medium to obtain a final concentration of 0.4 µM. Of this mixture, 25 μL were added to the corresponding cells in the well-plates followed by incubation of the cells for 1 h and 4 h, respectively, at 37 °C and 5% CO_2_. Afterwards, the tumor cells were rinsed three times with ice-cold PBS. To determine the internalized fraction, the AR42J, PC-3 PIP and KB tumor cells were shortly incubated (30 min, 10 min or just 2 min, respectively) with an acidic stripping buffer. In the case of AR42J cells and PC-3 PIP cells, the stripping buffer was a glycine/NaCl buffer of pH 2.8 while an aqueous solution of acetic acid/NaCl of pH 3.0 was used in the case of KB cells.

Trypsin (500 µL, Gibco^TM^ TrypLE^TM^ Express without phenol red; Fisher Scientific, Grand Island, NY, USA) was used to release the cells and transfer them to the microwave vials (MG5, 4 mL, Anton Paar Switzerland AG, Buchs, Switzerland). Holmium-165 (50 µL, 0.1 mg/L, in 2% (v/v) nitric acid) and concentrated nitric acid (650 µL, purified by redistillation, ≥99.999% trace metal basis, Merck KGaA, Darmstadt, Germany) were added to the cells in the microwave vials for subsequent microwave digestion at 150 °C for 10 min using a dedicated microwave system (Anton Paar Switzerland, Buchs, Switzerland). The digested cell samples were diluted by an ESI prepFAST precision M5X dilution system (Elemental Scientific, Inc. Ohama, NE, USA) and analyzed by a triple quadrupole ICP-MS (iCAP TQ, ThermoFisher, Reinach, Switzerland). Calibration curves for lutetium-175, holmium-165, terbium-159, gadolinium-157 and europium-153 were performed with freshly prepared metal dilutions ranging from 0.1 ng/L to 1.0 µg/L using standard solutions for ICP-MS (1 g/L solution in 2% (v/v) nitric acid Merck KGaA, Darmstadt, Germany).

***Results***: The results of the in vitro cell uptake studies of the Ln-labeled biomolecules are described and discussed in the main article.

**3. In vitro cell uptake studies of lanthanide chloride salts**

***Purpose***: In vitro cell uptake studies were also performed with lanthanide salts to investigate their cell uptake pattern.

***Methods***: The same experimental setting as described for Ln-labeled biomolecules was applied to perform uptake and internalization studies with the lanthanide salts (LnCl_3_; Ln = ^nat^Lu, ^nat^Tb, ^nat^Gd and ^nat^Eu). The applied solution was a mixture of the four lanthanide salts yielding a final concentration of 0.4 µM. Of these mixtures, 25 μL were added to the corresponding well with tumor cells. The cells were incubated for 1 h and 4 h, respectively, at 37 °C and 5% CO_2_. After incubation, the tumor cells were treated as described for the Ln-labeled biomolecules and processed for the measurement with ICP-MS.

***Results***: Variations in cell uptake after application of lanthanide chloride salts were observed irrespective of the tumor cell line. In all cell lines, the uptake of the lanthanides increased over the time of incubation. The amount of lanthanides detected in AR42J and PC-3 PIP cells was considerably lower after rinsing the cells with acidic glycine stripping buffer. The reduction in measured lanthanides after rinsing KB cells with an aqueous solution of acetic acid/NaCl was, however, only minimal. No significant difference was observed between lanthanide uptake when applied as [^159^Tb]TbCl_3_ and [^175^Lu]LuCl_3_ after incubation with AR42J and PC-3 PIP cells. After 1 h incubation with KB tumor cells, the detected amount of terbium was significantly lower (*p*<0.05) than that of lutetium after application of [^159^Tb]TbCl_3_ and [^175^Lu]LuCl_3_, respectively. After longer incubation periods, the difference between these two lanthanides was smaller. The fraction of gadolinium retained in the PC-3 PIP tumor cells after addition of the lanthanide salts and the acid-wash was significantly (*p*<0.05) ‬higher than that of lutetium at both investigated incubation times. On the contrary, in AR42J tumor cells, the detected fraction of gadolinium was significantly lower after acid-wash (*p*<0.05) than the detected fraction of lutetium after application of the lanthanide salts, after 1 h incubation. Only in AR42J tumor cells, a significantly (*p*

‬‬‬‬‬‬‬‬‬‬‬‬‬‬‬‬‬‬‬‬‬‬‬‬‬‬‬‬‬‬‬‬‬‬‬‬‬‬‬‬‬‬‬‬‬‬‬‬‬‬‬


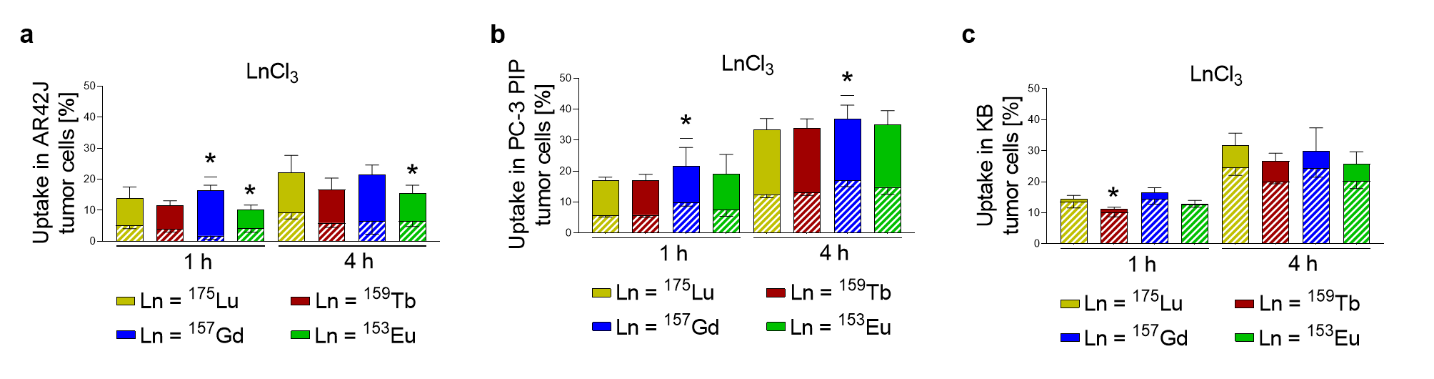


**Fig. S4** **a‒c** Uptake (entire bars) and internalization (hatched part of the bars) of lanthanides in **a** AR42J tumor cells, **b** PC-3 PIP tumor cells and **c** KB tumor cells. Data (average ± SD, n = 3-6) are presented in percent of the added lanthanide chloride salts (set as 100%). Asterisks indicate uptake (*) and internalization (*) values that are significantly different from the data obtained with the chloride salt of lutetium-175 (p<0.05)

**4. In vitro cell uptake studies of Ln-DTPA complexes**

***Purpose***: In vitro studies were also performed with lanthanide DTPA complexes to investigate their tumor cell uptake pattern.

***Methods***: The same experimental setting as described for Ln-labeled biomolecules was applied to perform uptake and internalization studies with the lanthanide DTPA complexes (Ln-DTPA; Ln = ^nat^Lu, ^nat^Tb, ^nat^Gd and ^nat^Eu). The lanthanides were dissolved in 0.05 M hydrochloric acid and further diluted in a 1:5 (v/v) mixture of sodium acetate (0.5 M) and hydrochloric acid (0.05 M), pH ~4.5. A 10-fold molar excess of DTPA (5 mM, aq.) was added to each lanthanide dilution and the reaction mixtures were incubated for 10 min at 95 °C. The complexed lanthanides were further diluted in saline with an adjusted pH value to 3.5-4.0 for in vitro experiments. The applied solution was a mixture of the four Ln-DTPA complexes at a final concentration of 0.4 µM. Of these mixtures, 25 μL were added to the well with the corresponding tumor cells. The tumor cells were incubated for 1 h and 4 h, respectively, at 37 °C and 5% CO_2_. After incubation, the tumor cells were treated as described for the Ln-labeled biomolecules and processed for the measurement with ICP-MS.

***Results***: The Ln-DTPA complexes did not bind to tumor cells and, hence, the detected lanthanides were within background levels (<20 ppt for lutetium-175, terbium-159 and europium-153 and <60 ppt for gadolinium-157) in samples of all tumor cell lines and at both investigated timepoints.

**5. In vivo biodistribution studies**

***Purpose***: The aim was to investigate the biodistribution of the Ln-labeled biomolecules and the lanthanides injected in chloride salt form or complexed with DTPA.

***Methods***: Biodistribution studies were performed with AR42J tumor-bearing mice after injection of Ln-DOTATATE and Ln-DOTA-LM3. Ln-PSMA-617 complexes were investigated in PC-3 PIP/flu tumor-bearing mice and Ln-OxFol-1 complexes in KB tumor-bearing mice. The biodistribution of the lanthanides after injection of chloride salts or complexed with DTPA was investigated in immunocompetent FVB mice. The experimental setup of the biodistribution studies is described in the main article.

ICP-MS analysis was performed as previously reported (Fig. S5) [3]. The collected organs were weighed and digested in the presence of a 9:1 (v/v) mixture of 69% (v/v) nitric acid and Milli-Q water (1.2 mL per 100 mg tissue). To correct the loss in the sample volume during the open-vessel microwave digestion approach and to correct potential matrix effects, holmium-165 (90 µL of a 0.1 mg/L solution in 2% (v/v) nitric acid) was added as internal standard before the digestion. Microwave digestion of the tissue was done at 150 °C for 10 min using a dedicated microwave system. The digested tissue samples were diluted by an ESI prepFAST precision M5X dilution system and directly introduced to a triple quadrupole ICP-MS system for analysis.

To determine the concentration of the lanthanides in the injection solution, 10 µL of this injection solution were diluted in 900 µL of a 2:1 (v/v) mixture of 69% (v/v) nitric acid and Milli-Q water. Holmium-165 (90 µL of a 0.1 mg/L solution in 2% (v/v) nitric acid) was added as internal standard before digestion at 150 °C for 10 min using microwaves. The lanthanide concentration was determined using ICP-MS to calculate the total amount of injected lanthanides, which was defined as 100%. Based on the obtained values, the percentage of lanthanide uptake in the specific organs and tissues was calculated and expressed as percent injected dose per gram tissue (% ID/g).

***Results***: Multiplexed ICP-MS analysis allowed the simultaneous detection of the stable isotopes of the four lanthanides, namely lutetium-175, terbium-159, gadolinium-157 and europium-153, in one animal (Fig. S5). The results of the biodistribution studies are described and discussed in the main article and presented in Tables S1-S12.


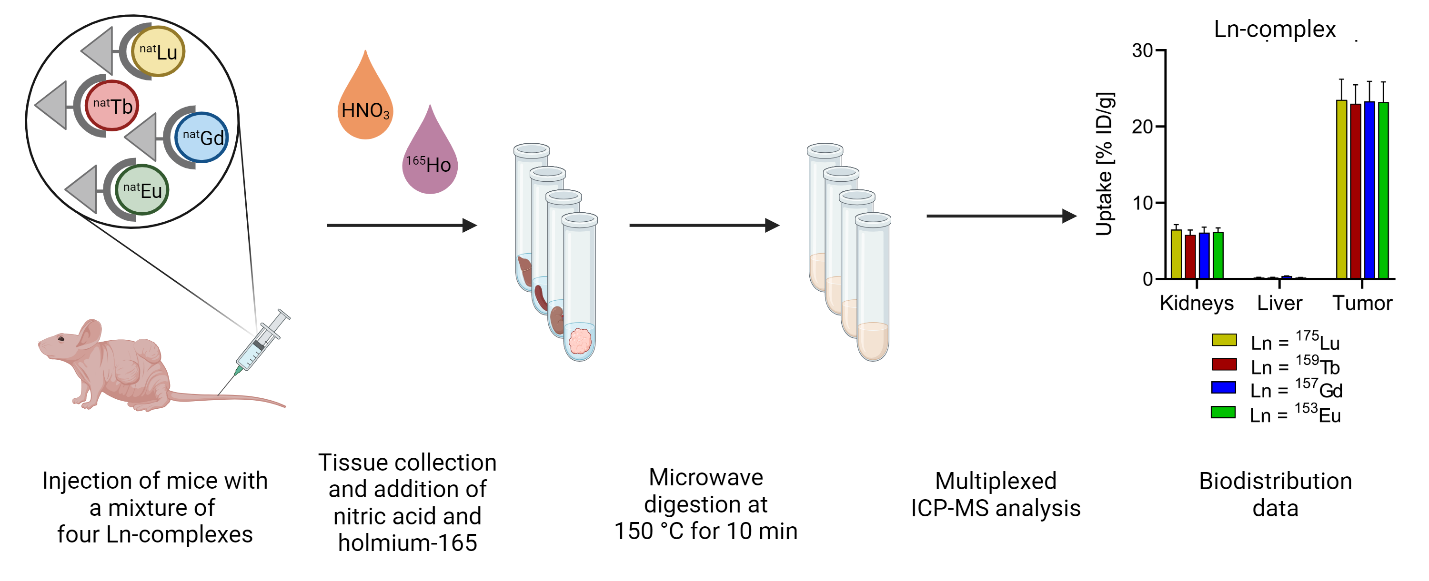


**Fig. S5** Schematic workflow of biodistribution studies of metal complexes prepared with naturally-occurring lanthanides (Ln-labeled biomolecules, LnCl_3_ or Ln-DTPA complexes), followed by multiplexed ICP-MS analysis. After injection of the animals with a mixture of Ln-complexes (Ln = ^nat^Lu, ^nat^Tb, ^nat^Gd and ^nat^Eu), the mice were sacrificed at specific time points and the collected tissues processed using microwave assisted nitric acid digestion. Multiplexed ICP-MS analysis of the processed tissues was performed for lutetium-175, terbium-159, gadolinium-157 and europium-153. (Figure created with BioRender.com)

**Table S1** Biodistribution data of Ln-DOTATATE at 1 h p.i. in AR42J tumor-bearing mice, shown as [% ID/g] values, representing the average ± SD of n=3 mice

|  | [^175^Lu]Lu-DOTATATE | [^159^Tb]Tb-DOTATATE | [^157^Gd]Gd-DOTATATE | [^153^Eu]Eu-DOTATATE |
| --- | --- | --- | --- | --- |
|  | n = 3 | n = 3 | n = 3 | n = 3 |
|  | [% ID/g] | [% ID/g] | [% ID/g] | [% ID/g] |
| Blood | <0.5 | <0.5 | <0.5 | <0.5 |
| Heart | <0.5 | <0.5 | 0.50 ± 0.07 | <0.5 |
| Lung | 0.77 ± 0.08 | 0.54 ± 0.09 | 0.79 ± 0.13 | 0.62 ± 0.07 |
| Spleen | <0.5 | <0.5 | 0.59 ± 0.06 | <0.5 |
| Kidneys | 6.5 ± 0.7 | 5.8 ± 0.6 | 6.1 ± 0.7 | 6.2 ± 0.5 |
| Stomach | 1.1 ± 0.1 | 0.79 ± 0.05 | 1.2 ± 0.1 | 0.81 ± 0.12 |
| Pancreas | 0.90 ± 0.04 | 0.72 ± 0.01 | 1.0 ± 0.2 | 0.74 ± 0.02 |
| Intestines | <0.5 | <0.5 | 0.53 ± 0.11 | <0.5 |
| Liver | <0.5 | <0.5 | <0.5 | <0.5 |
| Muscle | <0.5 | <0.5 | <0.5 | <0.5 |
| Bone | <0.5 | <0.5 | <0.5 | <0.5 |
| Salivary glands | <0.5 | <0.5 | <0.5 | <0.5 |
| Brain | <0.5 | <0.5 | <0.5 | <0.5 |
| AR42J tumor | 5.1 ± 0.9 | 4.1 ± 0.7 | 4.2 ± 0.9 | 3.8 ± 0.6 |
|  | AR42J tumor-to-background organ ratios | | | |
| Tumor-to-blood | >10^*^ | >8.3^*^ | >8.5^*^ | >7.6^*^ |
| Tumor-to-kidney | 0.80 ± 0.19 | 0.72 ± 0.13 | 0.70 ± 0.17 | 0.61 ± 0.12 |
| Tumor-to-liver | >10^*^ | >8.3^*^ | >8.5^*^ | >7.6^*^ |

^*^Estimated ratios as corresponding off-target organs have <0.5% ID/g lanthanide accumulation.

**Table S2** Biodistribution data of Ln-DOTATATE at 24 h p.i. in AR42J tumor-bearing mice, shown as [% ID/g] values, representing the average ± SD of n=3 mice

|  | [^175^Lu]Lu-DOTATATE | [^159^Tb]Tb-DOTATATE | [^157^Gd]Gd-DOTATATE | [^153^Eu]Eu-DOTATATE |
| --- | --- | --- | --- | --- |
|  | n = 3 | n = 3 | n = 3 | n = 3 |
|  | [% ID/g] | [% ID/g] | [% ID/g] | [% ID/g] |
| Blood | <0.5 | <0.5 | <0.5 | <0.5 |
| Heart | <0.5 | <0.5 | <0.5 | <0.5 |
| Lung | <0.5 | <0.5 | <0.5 | <0.5 |
| Spleen | <0.5 | <0.5 | 0.63 ± 0.19 | 1.2 ± 1.6 |
| Kidneys | 2.9 ± 0.8 | 2.7 ± 0.8 | 2.9 ± 0.9 | 2.8 ± 0.9 |
| Stomach | 0.57 ± 0.06 | <0.5 | 0.76 ± 0.11 | <0.5 |
| Pancreas | <0.5 | <0.5 | <0.5 | <0.5 |
| Intestines | <0.5 | <0.5 | <0.5 | <0.5 |
| Liver | <0.5 | <0.5 | <0.5 | <0.5 |
| Muscle | <0.5 | <0.5 | <0.5 | <0.5 |
| Bone | <0.5 | <0.5 | <0.5 | <0.5 |
| Salivary glands | <0.5 | <0.5 | <0.5 | <0.5 |
| Brain | <0.5 | <0.5 | <0.5 | <0.5 |
| AR42J tumor | 2.3 ± 0.3 | 1.8 ± 0.2 | 1.8 ± 0.2 | 1.7 ± 0.1 |
|  | AR42J tumor-to-background organ ratios | | | |
| Tumor-to-blood | >4.6^*^ | >3.7^*^ | >3.7^*^ | >3.4^*^ |
| Tumor-to-kidney | 0.86 ± 0.27 | 0.73 ± 0.24 | 0.69 ± 0.24 | 0.64 ± 0.20 |
| Tumor-to-liver^*^ | >4.6^*^ | >3.7^*^ | >3.7^*^ | >3.4^*^ |

^*^Estimated ratios as corresponding off-target organs have <0.5% ID/g lanthanide accumulation.

**Table S3** Biodistribution data of Ln-DOTA-LM3 at 1 h p.i. in AR42J tumor-bearing mice, shown as [% ID/g] values, representing the average ± SD of n=3 mice

|  | [^175^Lu]Lu-DOTA-LM3 | [^159^Tb]Tb-DOTA-LM3 | [^157^Gd]Gd-DOTA-LM3 | [^153^Eu]Eu-DOTA-LM3 |
| --- | --- | --- | --- | --- |
|  | n = 3 | n = 3 | n = 3 | n = 3 |
|  | [% ID/g] | [% ID/g] | [% ID/g] | [% ID/g] |
| Blood | 0.51 ± 0.10 | <0.5 | <0.5 | <0.5 |
| Heart | <0.5 | <0.5 | 0.73 ± 0.18 | <0.5 |
| Lung | 1.2 ± 0.1 | 1.2 ± 0.1 | 2.5 ± 1.8 | 2.2 ± 0.9 |
| Spleen | <0.5 | <0.5 | 0.79 ± 0.19 | 0.64 ± 0.17 |
| Kidneys | 11 ± 1 | 12 ± 2 | 12 ± 2 | 13 ± 2 |
| Stomach | 1.3 ± 0.2 | 1.1 ± 0.2 | 2.0 ± 0.7 | 1.3 ± 0.1 |
| Pancreas | 1.1 ± 0.1 | 1.2 ± 0.2 | 1.6 ± 0.2 | 1.3 ± 0.1 |
| Intestines | 0.60 ± 0.24 | <0.5 | 0.71 ± 0.28 | <0.5 |
| Liver | 1.2 ± 1.2 | 0.51 ± 0.04 | 0.53 ± 0.04 | 0.73 ± 0.07 |
| Muscle | <0.5 | <0.5 | 0.68 ± 0.15 | <0.5 |
| Bone | <0.5 | <0.5 | 0.57 ± 0.05 | 0.57 ± 0.06 |
| Salivary glands | <0.5 | <0.5 | 0.73 ± 0.12 | <0.5 |
| Brain | <0.5 | <0.5 | <0.5 | <0.5 |
| AR42J tumor | 4.5 ± 0.2 | 4.9 ± 0.3 | 4.9 ± 0.3 | 5.0 ± 0.4 |
|  | AR42J tumor-to-background organ ratios | | | |
| Tumor-to-blood | 9.1 ± 1.4 | 13 ± 1 | 10 ± 1 | 12 ± 2 |
| Tumor-to-kidney | 0.44 ± 0.07 | 0.43 ± 0.06 | 0.43 ± 0.06 | 0.40 ± 0.05 |
| Tumor-to-liver | 6.4 ± 4.2 | 9.6 ± 0.3 | 9.4 ± 0.6 | 6.9 ± 1.3 |

**Table S4** Biodistribution data of Ln-DOTA-LM3 at 24 h p.i. in AR42J tumor-bearing mice, shown as [% ID/g] values, representing the average ± SD of n=3 mice

|  | [^175^Lu]Lu-DOTA-LM3 | [^159^Tb]Tb-DOTA-LM3 | [^157^Gd]Gd-DOTA-LM3 | [^153^Eu]Eu-DOTA-LM3 |
| --- | --- | --- | --- | --- |
|  | n = 3 | n = 3 | n = 3 | n = 3 |
|  | [% ID/g] | [% ID/g] | [% ID/g] | [% ID/g] |
| Blood | <0.5 | <0.5 | <0.5 | <0.5 |
| Heart | <0.5 | <0.5 | 0.59 ± 0.19 | <0.5 |
| Lung | <0.5 | <0.5 | 0.52 ± 0.11 | <0.5 |
| Spleen | <0.5 | <0.5 | 0.68 ± 0.12 | <0.5 |
| Kidneys | 4.8 ± 0.5 | 5.2 ± 0.5 | 5.4 ± 0.77 | 5.4 ± 0.5 |
| Stomach | 0.76 ± 0.06 | 0.52 ± 0.03 | 1.3 ± 0.3 | 0.60 ± 0.05 |
| Pancreas | 0.51 ± 0.17 | <0.5 | 0.86 ± 0.19 | 0.54 ± 0.06 |
| Intestines | <0.5 | <0.5 | 0.57 ± 0.19 | <0.5 |
| Liver | <0.5 | <0.5 | <0.5 | <0.5 |
| Muscle | <0.5 | <0.5 | <0.5 | <0.5 |
| Bone | <0.5 | <0.5 | <0.5 | <0.5 |
| Salivary glands | <0.5 | <0.5 | 0.85 ± 0.71 | <0.5 |
| Brain | <0.5 | <0.5 | <0.5 | <0.5 |
| AR42J tumor | 2.7 ± 0.3 | 2.9 ± 0.3 | 3.1 ± 0.2 | 2.8 ± 0.3 |
|  | AR42J tumor-to-background organ ratios | | | |
| Tumor-to-blood | >5.4^*^ | >5.8^*^ | >6.2^*^ | >5.7^*^ |
| Tumor-to-kidney | 0.57 ± 0.11 | 0.57 ± 0.10 | 0.58 ± 0.11 | 0.54 ± 0.10 |
| Tumor-to-liver | >5.4^*^ | >5.8^*^ | >6.2^*^ | >5.7^*^ |

^*^Estimated ratios as corresponding off-target organs have <0.5% ID/g lanthanide accumulation.

**Table S5** Biodistribution data of Ln-PSMA-617 at 1 h p.i. in PC-3 PIP/flu tumor-bearing mice, shown as [% ID/g] values, representing the average ± SD of n=4 mice

|  | [^175^Lu]Lu-PSMA-617 | [^159^Tb]Tb-PSMA-617 | [^157^Gd]Gd-PSMA-617 | [^153^Eu]Eu-PSMA-617 |
| --- | --- | --- | --- | --- |
|  | n = 4 | n = 4 | n = 4 | n = 4 |
|  | [% ID/g] | [% ID/g] | [% ID/g] | [% ID/g] |
| Blood | <0.5 | <0.5 | <0.5 | <0.5 |
| Heart | <0.5 | <0.5 | 0.83 ± 0.31 | 0.52 ± 0.42 |
| Lung | <0.5 | <0.5 | 0.71 ±0.15 | <0.5 |
| Spleen | 0.64 ± 0.48 | <0.5 | 0.92 ± 0.30 | 0.51 ± 0.17 |
| Kidneys | 10 ± 4 | 8.4 ± 3.5 | 8.4 ± 3.4 | 8.1 ± 3.5 |
| Stomach | 0.56 ± 0.19 | <0.5 | 1.2 ± 0.2 | <0.5 |
| Intestines | <0.5 | <0.5 | 0.60 ± 0.15 | <0.5 |
| Liver | <0.5 | <0.5 | 0.63 ± 0.11 | <0.5 |
| Muscle | <0.5 | <0.5 | 0.71 ± 0.10 | <0.5 |
| Bone | <0.5 | <0.5 | <0.5 | <0.5 |
| Salivary glands | <0.5 | <0.5 | 0.86 ± 0.23 | <0.5 |
| PC-3 PIP tumor | 25 ± 3 | 25 ± 3 | 25 ± 3 | 24 ± 3 |
| PC-3 flu tumor | <0.5 | <0.5 | 0.61 ± 0.38 | <0.5 |
|  | PC-3 PIP tumor-to-background organ ratios | | | |
| Tumor-to-blood | >51^*^ | >51^*^ | >51^*^ | >50^*^ |
| Tumor-to-kidney | 2.7 ± 0.8 | 3.3 ± 1.0 | 3.3 ± 1.1 | 3.3 ± 1.1 |
| Tumor-to-liver | >51^*^ | >51^*^ | >40^*^ | >50^*^ |

^*^Estimated ratios as corresponding off-target organs have <0.5% ID/g lanthanide accumulation.

**Table S6** Biodistribution data of Ln-PSMA-617 at 4 h p.i. in PC-3 PIP/flu tumor-bearing mice, shown as [% ID/g] values, representing the average ± SD of n=4 mice

|  | [^175^Lu]Lu-PSMA-617 | [^159^Tb]Tb-PSMA-617 | [^157^Gd]Gd-PSMA-617 | [^153^Eu]Eu-PSMA-617 |
| --- | --- | --- | --- | --- |
|  | n = 4 | n = 4 | n = 4 | n = 4 |
|  | [% ID/g] | [% ID/g] | [% ID/g] | [% ID/g] |
| Blood | <0.5 | <0.5 | <0.5 | <0.5 |
| Heart | <0.5 | <0.5 | 0.61 ± 0.11 | <0.5 |
| Lung | <0.5 | <0.5 | <0.5 | <0.5 |
| Spleen | <0.5 | <0.5 | 0.78 ± 0.08 | <0.5 |
| Kidneys | 1.4 ± 0.4 | 1.0 ± 0.2 | 1.1 ± 0.2 | 1.1 ± 0.3 |
| Stomach | <0.5 | <0.5 | 1.1 ± 0.2 | <0.5 |
| Intestines | <0.5 | <0.5 | <0.5 | <0.5 |
| Liver | <0.5 | <0.5 | <0.5 | <0.5 |
| Muscle | <0.5 | <0.5 | 0.55 ± 0.08 | <0.5 |
| Bone | <0.5 | <0.5 | <0.5 | <0.5 |
| Salivary glands | <0.5 | <0.5 | 0.53 ± 0.09 | <0.5 |
| PC-3 PIP tumor | 23 ± 3 | 23 ± 3 | 23 ± 3 | 23 ± 3 |
| PC-3 flu tumor | <0.5 | <0.5 | 0.68 ± 0.28 | <0.5 |
|  | PC-3 PIP tumor-to-background organ ratios | | | |
| Tumor-to-blood | >47^*^ | >46^*^ | >47^*^ | >46^*^ |
| Tumor-to-kidney | 18 ± 3 | 24 ± 3 | 21 ± 2 | 23 ± 3 |
| Tumor-to-liver | >47^*^ | >46^*^ | >43^*^ | >46^*^ |

^*^Estimated ratios as corresponding off-target organs have <0.5% ID/g lanthanide accumulation.

**Table S7** Biodistribution data of Ln-OxFol-1 at 4 h p.i. in KB tumor-bearing mice, shown as [% ID/g] values, representing the average ± SD of n=4 mice

|  | [^175^Lu]Lu-OxFol-1 | [^159^Tb]Tb-OxFol-1 | [^157^Gd]Gd-OxFol-1 | [^153^Eu]Eu-OxFol-1 |
| --- | --- | --- | --- | --- |
|  | n = 4 | n = 4 | n = 4 | n = 4 |
|  | [% ID/g] | [% ID/g] | [% ID/g] | [% ID/g] |
| Blood | 11 ± 2 | 12 ± 2 | 12 ± 2 | 9.9 ± 1.7 |
| Heart | 4.2 ± 0.5 | 5.0 ± 0.3 | 5.3 ± 0.5 | 4.3 ± 0.4 |
| Lung | 8.7 ± 2.9 | 7.2 ± 1.2 | 7.6 ± 1.3 | 7.0 ± 1.5 |
| Spleen | 5.7 ± 1.2 | 2.8 ± 0.5 | 5.0 ± 0.6 | 6.0 ± 1.1 |
| Kidneys | 35 ± 5 | 43 ± 6 | 41 ± 5 | 34 ± 4.3 |
| Stomach | 1.9 ± 0.4 | 2.4 ± 0.5 | 2.8 ± 0.4 | 2.0 ± 0.4 |
| Intestines | 1.7 ± 0.3 | 2.1 ± 0.4 | 2.2 ± 0.4 | 1.7 ± 0.3 |
| Liver | 6.8 ± 0.9 | 4.3 ± 0.6 | 6.3 ± 0.9 | 7.5 ± 1.1 |
| Muscle | 1.6 ± 0.2 | 2.0 ± 0.3 | 2.3 ± 0.5 | 1.6 ± 0.3 |
| Bone | 1.6 ± 0.3 | 1.6 ± 0.3 | 1.7 ± 0.3 | 1.5 ± 0.3 |
| Brain | 0.60 ± 0.05 | 0.73 ± 0.08 | 0.83 ± 0.09 | 0.58 ± 0.06 |
| Salivary glands | 5.8 ± 0.5 | 7.6 ± 0.9 | 7.4 ± 0.8 | 5.9 ± 0.5 |
| KB tumor | 22 ± 4 | 28 ± 5 | 27 ± 5 | 22 ± 4 |
|  | KB tumor-to-background organ ratios | | | |
| Tumor-to-blood | 2.1 ± 1 | 2.4 ±1 | 2.3 ± 1 | 2.3 ± 1 |
| Tumor-to-kidney | 0.64 ± 0.17 | 0.67 ± 0.17 | 0.66 ± 0.17 | 0.66 ± 0.18 |
| Tumor-to-liver | 3.2 ± 0.9 | 6.6 ± 1.8 | 4.3 ± 1.0 | 3.0 ± 0.7 |

**Table S8** Biodistribution data of Ln-OxFol-1 at 24 h p.i. in KB tumor-bearing mice, shown as [% ID/g] values, representing the average ± SD of n=4 mice

|  | [^175^Lu]Lu-OxFol-1 | [^159^Tb]Tb-OxFol-1 | [^157^Gd]Gd-OxFol-1 | [^153^Eu]Eu-OxFol-1 |
| --- | --- | --- | --- | --- |
|  | n = 4 | n = 4 | n = 4 | n = 4 |
|  | [% ID/g] | [% ID/g] | [% ID/g] | [% ID/g] |
| Blood | <0.5 | <0.5 | 0.54 ± 0.02 | <0.5 |
| Heart | 1.7 ± 0.1 | 2.1 ± 0.4 | 2.3 ± 0.2 | 1.8 ± 0.3 |
| Lung | 2.0 ± 0.4 | 2.2 ± 0.3 | 2.6 ± 0.5 | 2.3 ± 0.6 |
| Spleen | 4.3 ± 1.0 | 1.6 ± 0.4 | 3.5 ± 1.2 | 4.7 ± 1.2 |
| Kidneys | 38 ± 1 | 42 ± 2 | 41 ± 2 | 37 ± 1 |
| Stomach | 1.1 ± 0.2 | 1.6 ± 0.3 | 2.1 ± 0.3 | 1.6 ± 0.3 |
| Intestines | <0.5 | 0.50 ± 0.09 | 0.77 ± 0.10 | 0.54 ± 0.10 |
| Liver | 5.9 ± 0.7 | 3.3 ± 0.5 | 4.9 ± 0.9 | 6.4 ± 0.9 |
| Muscle | 1.3 ± 0.3 | 1.6 ± 0.4 | 2.0 ± 0.5 | 1.5 ± 0.4 |
| Bone | 0.88 ± 0.09 | 0.85 ± 0.06 | 1.1 ± 0.1 | 0.99 ± 0.09 |
| Brain | 0.64 ± 0.02 | 0.74 ± 0.05 | 0.84 ± 0.03 | 0.66 ± 0.03 |
| Salivary glands | 4.3 ± 0.9 | 5.0 ± 0.9 | 5.3 ± 0.7 | 4.6 ± 0.7 |
| KB tumor | 28 ± 6 | 31 ± 7 | 30 ± 7 | 28 ± 6 |
|  | KB tumor-to-background organ ratios | | | |
| Tumor-to-blood | >56^*^ | >63^*^ | 57 ± 13 | >56^*^ |
| Tumor-to-kidney | 0.73 ± 0.17 | 0.75 ± 0.20 | 0.75 ± 0.18 | 0.75 ± 0.18 |
| Tumor-to-liver | 4.8 ± 1.1 | 9.8 ± 3.1 | 6.4 ± 2.0 | 4.5 ± 1.3 |

^*^Estimated ratios as corresponding off-target organ has <0.5% ID/g lanthanide accumulation.

**Table S9** Biodistribution data of LnCl_3_ at 1 h p.i. in non-tumor-bearing mice, shown as [% ID/g] values, representing the average ± SD of n=3 mice

|  | [^175^Lu]LuCl_3_ | [^159^Tb]TbCl_3_ | [^157^Gd]GdCl_3_ | [^153^Eu]EuCl_3_ |
| --- | --- | --- | --- | --- |
|  | n = 3 | n = 3 | n = 3 | n = 3 |
|  | [% ID/g] | [% ID/g] | [% ID/g] | [% ID/g] |
| Blood | 6.4 ± 2.7 | 5.0 ± 0.9 | 4.9 ± 0.3 | 5.5 ± 0.6 |
| Heart | 3.6 ± 1.5 | 4.8 ± 2.2 | 5.0 ± 1.7 | 5.3 ± 2.1 |
| Lung | 5.6 ± 2.2 | 4.8 ± 1.2 | 4.8 ± 0.4 | 4.8 ± 1.1 |
| Spleen | 3.8 ± 0.5 | 6.1 ± 1.4 | 6.3 ± 0.9 | 6.9 ± 1.6 |
| Kidneys | 7.3 ± 1.7 | 8.7 ± 2.5 | 8.0 ± 2.0 | 8.1 ± 2.1 |
| Stomach | 5.8 ± 2.1 | 6.0 ± 2.3 | 6.2 ± 1.7 | 6.0 ± 2.3 |
| Pancreas | 2.6 ± 0.8 | 2.2 ± 1.0 | 2.2 ± 0.4 | 2.2 ± 1.0 |
| Intestines | 3.2 ± 1.1 | 2.6 ± 1.0 | 2.5 ± 0.8 | 2.6 ± 0.9 |
| Liver | 11 ± 3 | 27 ± 3 | 34 ± 4 | 41 ± 5 |
| Muscle | 1.5 ± 0.6 | 1.6 ± 0.9 | 1.6 ± 0.6 | 1.7 ± 1.0 |
| Bone | 18 ± 3 | 13 ± 2 | 13 ± 1 | 14 ± 2 |
| Salivary glands | 2.6 ± 0.9 | 2.4 ± 1.4 | 2.8 ± 1.3 | 2.7 ± 1.7 |
| Brain | <0.5 | 1.1 ± 0.8 | 1.0 ± 0.6 | 1.2 ± 0.9 |
|  | Organ ratios | | | |
| Blood-to-bone | 0.38 ± 0.23 | 0.38 ± 0.08 | 0.37 ± 0.01 | 0.40 ± 0.05 |
| Blood-to-liver | 0.61 ± 0.35 | 0.19 ± 0.05 | 0.14 ± 0.02 | 0.14 ± 0.02 |
| Liver-to-bone | 0.64 ± 0.13 | 2.0 ± 0.3 | 2.6 ± 0.4 | 3.0 ± 0.4 |

**Table S10** Biodistribution data of LnCl_3_ at 24 h p.i. in non-tumor-bearing mice, shown as [% ID/g] values, representing the average ± SD of n=3 mice

|  | [^175^Lu]LuCl_3_ | [^159^Tb]TbCl_3_ | [^157^Gd]GdCl_3_ | [^153^Eu]EuCl_3_ |
| --- | --- | --- | --- | --- |
|  | n = 3 | n = 3 | n = 3 | n = 3 |
|  | [% ID/g] | [% ID/g] | [% ID/g] | [% ID/g] |
| Blood | <0.5 | <0.5 | <0.5 | <0.5 |
| Heart | 0.54 ± 0.06 | 0.76 ± 0.10 | 1.2 ± 0.1 | 1.0 ± 0.1 |
| Lung | 0.88 ± 0.13 | 1.0 ± 0.1 | 1.3 ± 0.1 | 1.1 ± 0.2 |
| Spleen | 3.5 ± 0.9 | 5.0 ± 1.1 | 5.7 ± 1.1 | 5.6 ± 1.1 |
| Kidneys | 3.7 ± 1.0 | 3.8 ± 0.8 | 4.0 ± 0.9 | 3.8 ± 0.9 |
| Stomach | 1.8 ± 0.3 | 1.8 ± 0.4 | 2.6 ± 0.1 | 1.9 ± 0.5 |
| Pancreas | <0.5 | <0.5 | 0.61 ± 0.11 | <0.5 |
| Intestines | 0.61 ± 0.02 | 0.54 ± 0.05 | 0.71 ± 0.04 | 0.58 ± 0.06 |
| Liver | 12 ± 3 | 22 ± 5 | 31 ± 6 | 37 ± 7 |
| Muscle | <0.5 | <0.5 | 1.1 ± 0.8 | <0.5 |
| Bone | 25 ± 5 | 15 ± 2 | 14 ± 2 | 14 ± 3 |
| Salivary glands | 0.55 ± 0.17 | <0.5 | 0.77 ± 0.16 | 0.53 ± 0.17 |
| Brain | <0.5 | <0.5 | <0.5 | <0.5 |
|  | Organ ratios | | | |
| Blood-to-bone | <0.02^*^ | <0.04^*^ | <0.03^*^ | <0.04^*^ |
| Blood-to-liver | <0.05^*^ | <0.02^*^ | <0.02^*^ | <0.01^*^ |
| Liver-to-bone | 0.49 ± 0.19 | 1.6 ± 0.5 | 2.2 ± 0.5 | 2.7 ± 0.8 |

^*^Estimated ratios as corresponding off-target organs have <0.5% ID/g lanthanide accumulation.

**Table S11** Biodistribution data of LnCl_3_ at 96 h p.i. in non-tumor-bearing mice, shown as [% ID/g] values, representing the average ± SD of n=3 mice

|  | [^175^Lu]LuCl_3_ | [^159^Tb]TbCl_3_ | [^157^Gd]GdCl_3_ | [^153^Eu]EuCl_3_ |
| --- | --- | --- | --- | --- |
|  | n = 3 | n = 3 | n = 3 | n = 3 |
|  | [% ID/g] | [% ID/g] | [% ID/g] | [% ID/g] |
| Blood | <0.5 | <0.5 | <0.5 | <0.5 |
| Heart | 0.89 ± 0.25 | 0.73 ± 0.05 | 1.2 ± 0.1 | 0.83 ± 0.05 |
| Lung | 1.1 ± 0.2 | 0.91 ± 0.10 | 1.1 ± 0.2 | 0.88 ± 0.15 |
| Spleen | 2.3 ± 0.2 | 3.1 ± 0.2 | 3.6 ± 0.3 | 3.5 ± 0.3 |
| Kidneys | 2.7 ± 0.2 | 2.0 ± 0.1 | 2.0 ± 0.1 | 1.9 ± 0.1 |
| Stomach | 2.5 ± 0.2 | 2.2 ± 0.4 | 3.1 ± 0.8 | 2.3 ± 0.3 |
| Pancreas | <0.5 | <0.5 | 0.53 ± 0.10 | <0.5 |
| Intestines | 0.54 ± 0.14 | 0.52 ± 0.01 | 0.66 ± 0.07 | 0.53 ± 0.04 |
| Liver | 4.8 ± 0.9 | 11 ± 1 | 18 ± 1 | 23 ± 1 |
| Muscle | <0.5 | <0.5 | 0.51 ± 0.12 | <0.5 |
| Bone | 35 ± 4 | 19 ± 4 | 17 ± 3 | 18 ± 3 |
| Salivary glands | 0.50 ± 0.06 | <0.5 | 0.60 ± 0.08 | <0.5 |
| Brain | <0.5 | <0.5 | <0.5 | <0.5 |
|  | Organ ratios | | | |
| Blood-to-bone | <0.01^*^ | <0.03^*^ | <0.03^*^ | <0.03^*^ |
| Blood-to-liver | <0.11^*^ | <0.05^*^ | <0.03^*^ | <0.02^*^ |
| Liver-to-bone | 0.14 ± 0.04 | 0.59 ± 0.18 | 1.1 ± 0.2 | 1.3 ± 0.2 |

^*^Estimated ratios as corresponding off-target organs have <0.5% ID/g lanthanide accumulation.

**Table S12** Biodistribution data of Ln-DTPA at 1 h p.i. in non-tumor-bearing mice, shown as [% ID/g] values, representing the average ± SD of n=3 mice

|  | [^175^Lu]Lu-DTPA | [^159^Tb]Tb-DTPA | [^157^Gd]Gd-DTPA | [^153^Eu]Eu-DTPA |
| --- | --- | --- | --- | --- |
|  | n = 3 | n = 3 | n = 3 | n = 3 |
|  | [% ID/g] | [% ID/g] | [% ID/g] | [% ID/g] |
| Blood* | <0.5 | <0.5 | <0.5 | <0.5 |
| Heart | <0.5 | <0.5 | 0.55 ± 0.06 | <0.5 |
| Lung | <0.5 | <0.5 | 0.56 ± 0.12 | <0.5 |
| Spleen | <0.5 | <0.5 | 0.52 ± 0.03 | <0.5 |
| Kidneys | 2.3 ± 1.0 | 2.1 ± 1.0 | 2.1 ± 0.9 | 2.1 ± 1.0 |
| Stomach | <0.5 | <0.5 | 0.77 ± 0.17 | 0.51 ± 0.07 |
| Pancreas | <0.5 | <0.5 | <0.5 | <0.5 |
| Intestines | <0.5 | <0.5 | <0.5 | <0.5 |
| Liver^*^ | <0.5 | <0.5 | <0.5 | <0.5 |
| Muscle | <0.5 | <0.5 | 0.51 ± 0.07 | <0.5 |
| Bone^*^ | <0.5 | <0.5 | <0.5 | <0.5 |
| Salivary glands | <0.5 | <0.5 | <0.5 | <0.5 |
| Brain | <0.5 | <0.5 | <0.5 | <0.5 |

^*^Organ ratios were not calculated as corresponding organs have <0.5% ID/g lanthanide accumulation.

**6. Radiolabeling of the biomolecules**

***Purpose***: DOTATATE, DOTA-LM3, PSMA-617 and OxFol-1 were radiolabeled with lutetium-177 and terbium-161 to visualize their tissue distribution using the methodology of dual-isotope SPECT/CT imaging.

***Methods***: *Somatostatin analogues:* The stock solutions of 1 mM peptide concentration as described above were used for the preparation of radiolabeled DOTATATE and DOTA-LM3. Lutetium-177 (no-carrier-added [^177^Lu]LuCl_3_ in 0.04 M hydrochloric acid) was obtained from ITM Medical Isotopes GmbH, Munich, Germany. Terbium-161 (no-carrier-added [^161^Tb]TbCl_3_ in 0.05 M hydrochloric acid) was provided from in-house production by the “Radionuclide Development” Group at the PSI. DOTATATE and DOTA-LM3 were labeled with lutetium-177 and terbium-161 at a molar activity of 20 MBq/nmol under standard labeling conditions at pH ~4.5, using a 1:5 (v/v) mixture of sodium acetate (0.5 M) and hydrochloric acid (0.05 M) as previously reported [4]. The reaction mixtures were incubated for 10 min at 95 °C.

*PSMA ligand*: A stock solution of 1 mM ligand concentration as described above was used for the radiolabeling of PSMA-617 with lutetium-177 and terbium-161 at a molar activity of 20 MBq/nmol as previously reported [5] and described for the somatostatin analogues.

*Folate conjugate*: A stock solution of 1 mM ligand concentration as described above was used for the radiolabeling of OxFol-1 with lutetium-177 and terbium-161 at a molar activity of 20 MBq/nmol as previously reported [2] and described for the somatostatin analogues.

The quality of the radioconjugates was assessed as previously reported [2, 4, 5]. An aliquot of the radioconjugate was diluted in Na_5_-DTPA (50 μM, aq.). The sample was analyzed using a Merck Hitachi LaChrom high-performance liquid chromatography (HPLC) system consisting of an L-7100 pump, a D-700 interface, an L-7200 autosampler and a radioactivity detector (LB 506 B, Berthold Technologies GmbH) connected with a reversed-phase C-18 column (Xterra^TM^ MS, C18, 5 μm, 150 × 4.6 mm; Waters). A linear gradient of Milli-Q water containing 0.1% trifluoroacetic acid (95‒20%) and acetonitrile (5‒80%) over 15 min was applied at a flow rate of 1 mL/min.

***Results***: HPLC analysis of the ^177^Lu- and ^161^Tb-labeled biomolecules revealed high radiochemical purity (>99%) so that they could be used without further purification steps.

**7. Dual-isotope SPECT/CT imaging**

***Purpose***: Tissue distribution of ^177^Lu- and ^161^Tb-labeled biomolecules injected as a mixture was investigated using dual-isotope SPECT/CT imaging methodology. In addition, the tissue distribution profiles of lutetium-177 and terbium-161 injected in salt form were simultaneously visualized.

***Methods:*** DTPA (5mM) was added after radiolabeling of the biomolecules to complex unreacted radiometals. This was followed by the addition of L-ascorbic acid (3 mg) to prevent oxidation and additional sodium acetate (60 µL of 0.5M, pH 8 solution) to obtain a pH of 4.0-4.5. The solutions were then diluted (20 MBq/100 µL) in saline containing 0.05% BSA. The resulting solutions of each biomolecule labeled with lutetium-177 (10 MBq; 0.5 nmol) and terbium-161 (10 MBq, 0.5 nmol) were mixed in equal volumes to prepare the injection solutions. Injection solutions prepared for ^177^Lu- and ^161^Tb-labeled DOTATATE, DOTA-LM3 and OxFol-1 were administered into AR42J and KB tumor-bearing CD1 nude mice, respectively, while the injection solution of ^177^Lu- and ^161^Tb-labeled PSMA-617 was administered into PC-3 PIP/flu tumor-bearing athymic nude mice.

The injection formulation of [^177^Lu]LuCl_3_ and [^161^Tb]TbCl_3_ were prepared by using a 1:5 (v/v) mixture of sodium acetate (0.5 M) and hydrochloric acid (0.05 M), followed by dilution of the solutions (20 MBq/100 µL) in saline containing 0.05% BSA. The pH of the final injection solution was approximately 4.5. In order to apply lutetium-177 and terbium-161 as colloids, their respective chloride salts were diluted (20 MBq/100 µL) in PBS pH 7.4 and left to stand for 20 min. The injection solutions were prepared by mixing equal volumes of the acidic or neutral dilutions, which were applied on non-tumor-bearing FVB mice.

***Results:*** The results of the dual-isotope SPECT/CT studies are described and discussed in the main article and shown for two representative mice of each injection solution in the figures below (Figs. S6‒S11).


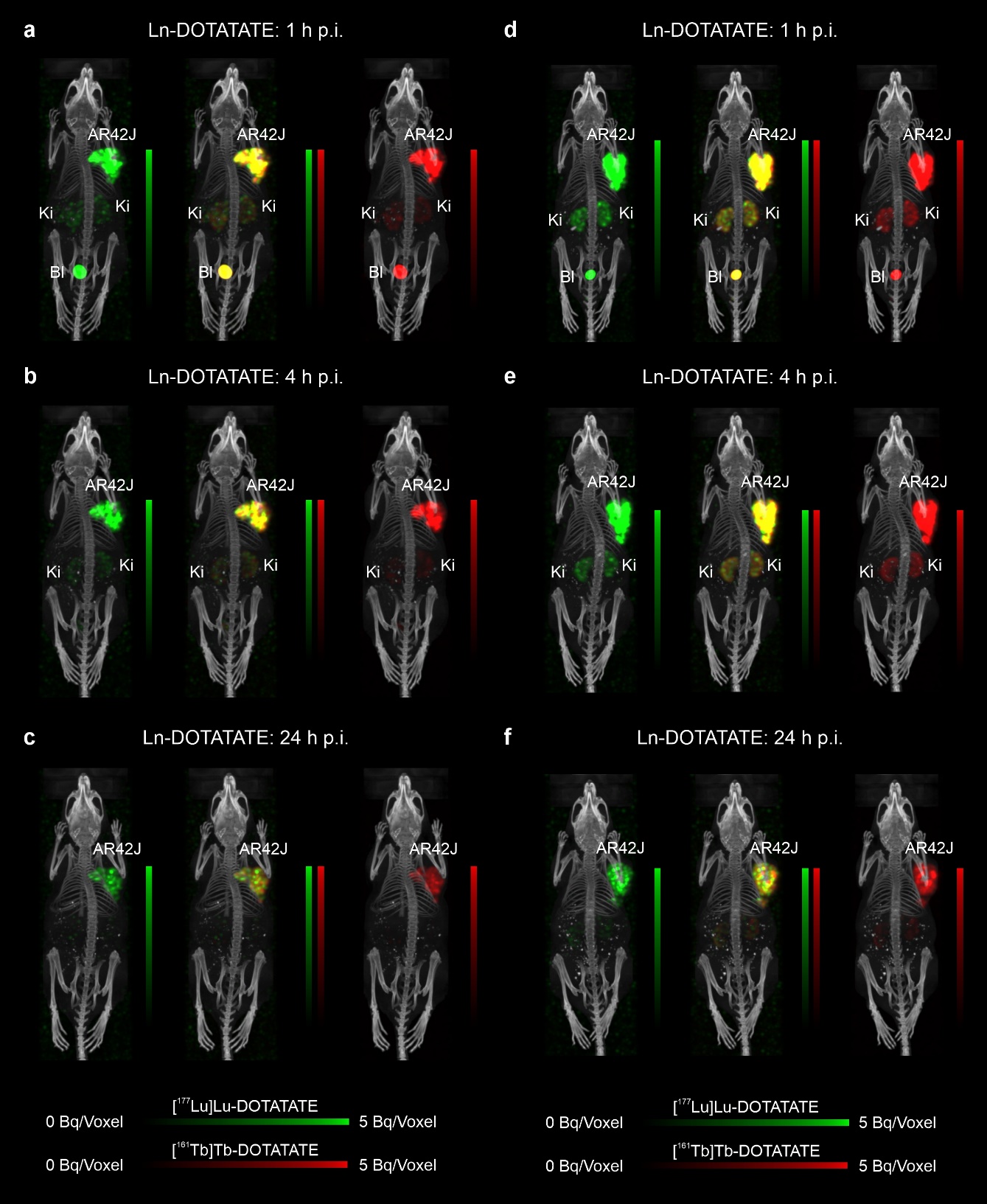


**Fig. S6** **a‒f** Dual-isotope SPECT/CT images of two representative AR42J tumor-bearing nude mice (**a‒c** and **d‒e**) injected with a mixture of [^177^Lu]Lu-DOTATATE (10 MBq, 0.5 nmol) and [^161^Tb]Tb-DOTATATE (10 MBq, 0.5 nmol); **a/d** SPECT/CT images at 1 h p.i.; **b/e** SPECT/CT images at 4 h p.i.; **c/f** SPECT/CT images at 24 h p.i.; (AR42J, tumor xenograft; Ki, kidney; Bl, urinary bladder)

**
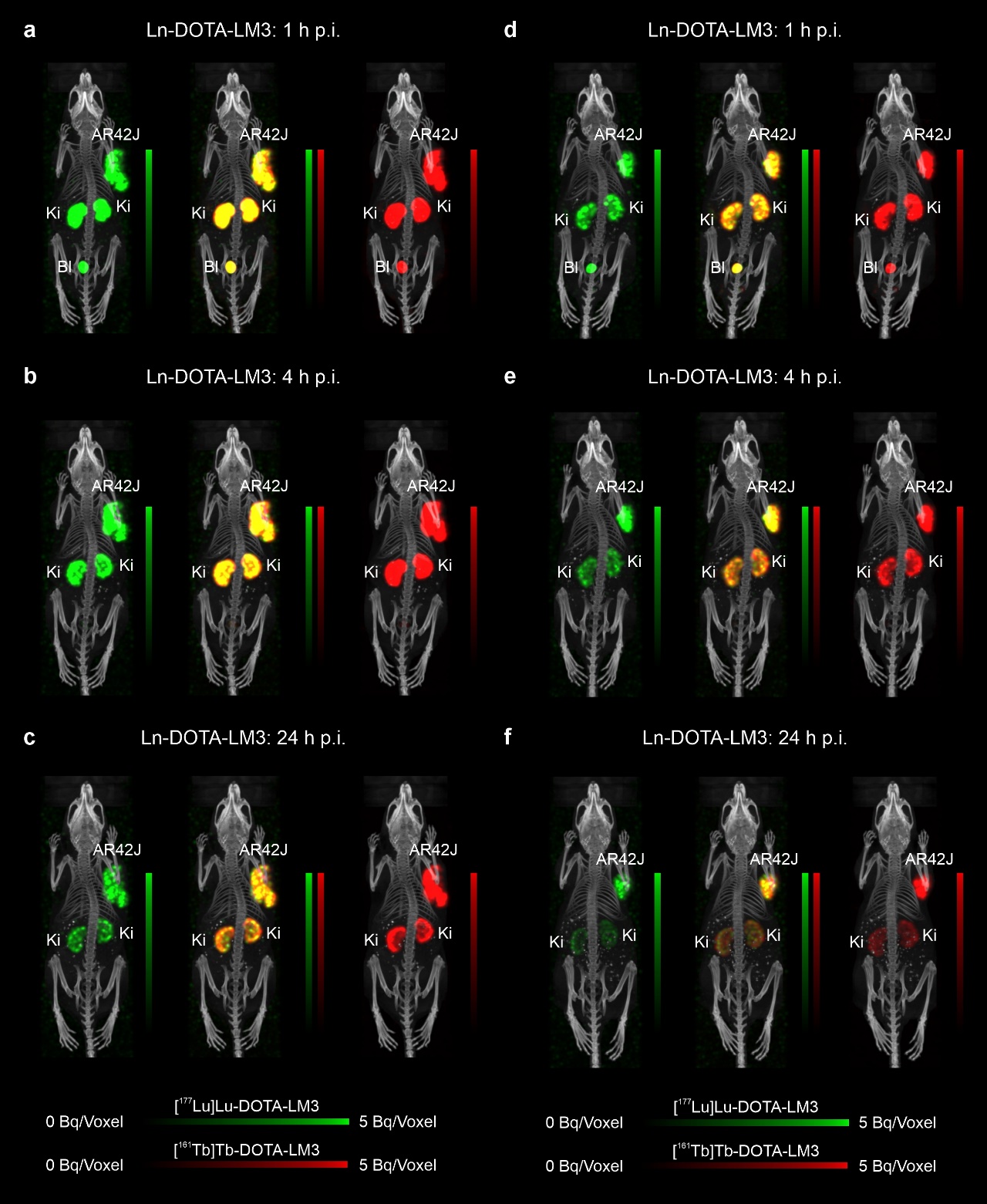
**

**Fig. S7** **a‒f** Dual-isotope SPECT/CT images of two representative AR42J tumor-bearing nude mice (**a‒c** and **d‒e**) injected with a mixture of [^177^Lu]Lu-DOTA-LM3 (10 MBq, 0.5 nmol) and [^161^Tb]Tb-DOTA-LM3 (10 MBq, 0.5 nmol); **a/d** SPECT/CT images at 1 h p.i.; **b**/**e** SPECT/CT images at 4 h p.i.; **c/f** SPECT/CT images at 24 h p.i.; (AR42J, tumor xenograft; Ki, kidney; Bl, urinary bladder)


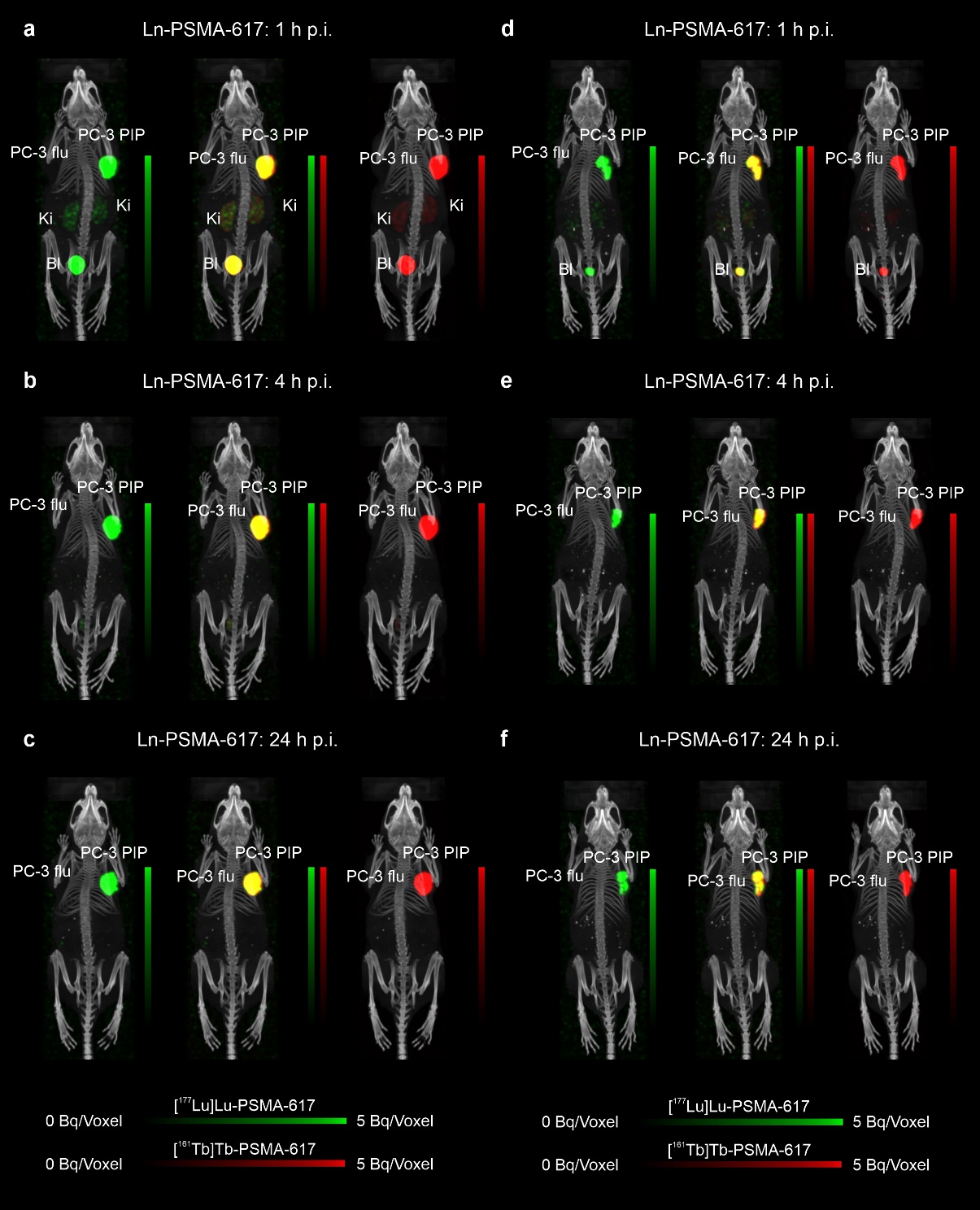


**Fig. S8** **a‒f** Dual-isotope SPECT/CT images of two representative PC-3 PIP/flu tumor-bearing nude mice (**a‒c** and **d‒e**) injected with a mixture of [^177^Lu]Lu-PSMA-617 (10 MBq, 0.5 nmol) and [^161^Tb]Tb-PSMA-617 (10 MBq, 0.5 nmol); **a/d** SPECT/CT images at 1 h p.i.; **b/e** SPECT/CT images at 4 h p.i.; **c/f** SPECT/CT images at 24 h p.i.; (PC-3 PIP, PSMA-positive tumor xenograft; PC-3 flu, PSMA-negative tumor xenograft; Ki, kidney; Bl, urinary bladder)


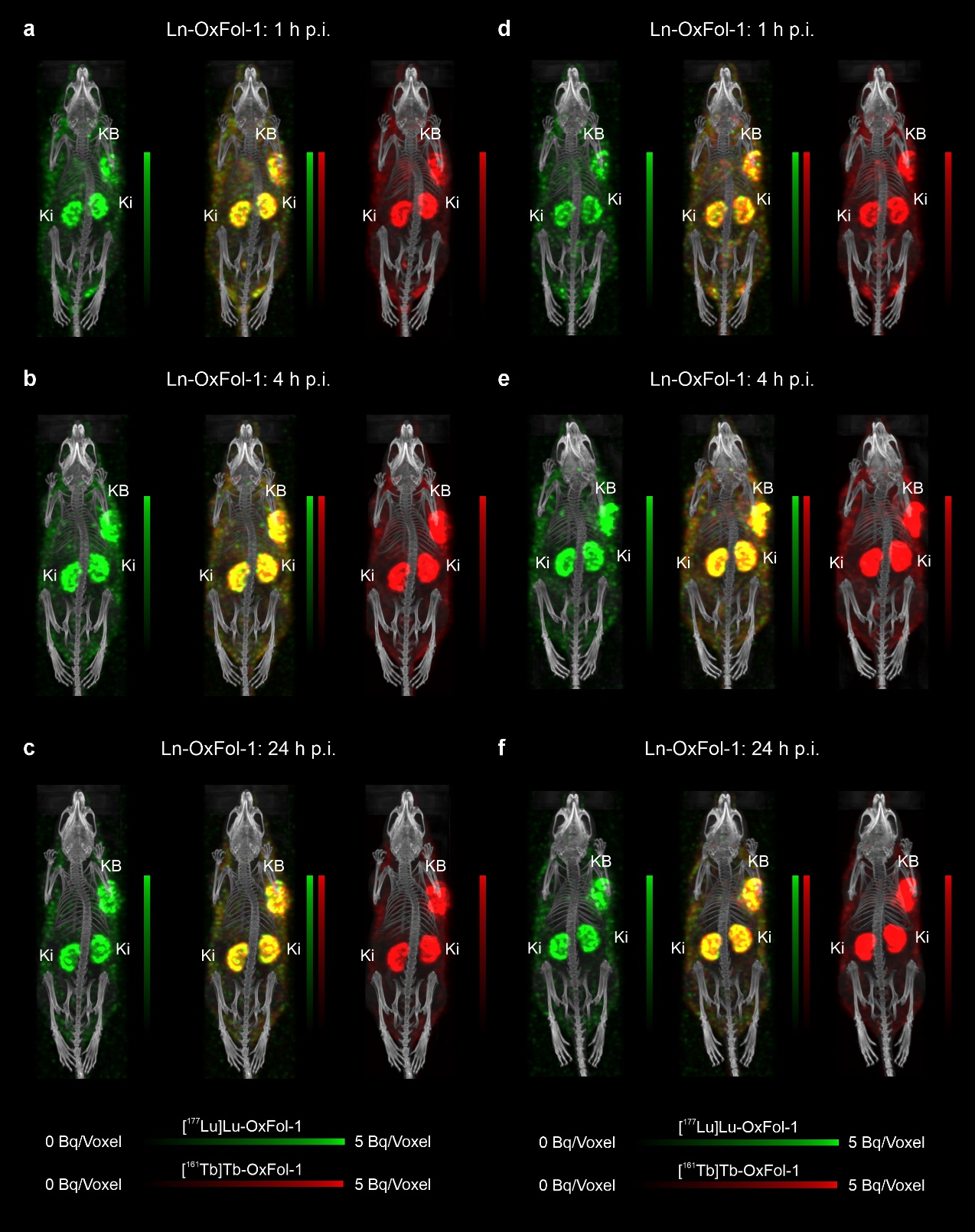


**Fig. S9** **a‒f** Dual-isotope SPECT/CT images of two representative KB tumor-bearing nude mice (**a‒c** and **d‒e**) injected with a mixture of [^177^Lu]Lu-OxFol-1 (10 MBq, 0.5 nmol) and [^161^Tb]Tb-OxFol-1 (10 MBq, 0.5 nmol); **a/d** SPECT/CT images at 1 h p.i.; **b/e** SPECT/CT images at 4 h p.i.; **c/f** SPECT/CT images at 24 h p.i.; (KB, FR-positive tumor xenograft; Ki, kidney)


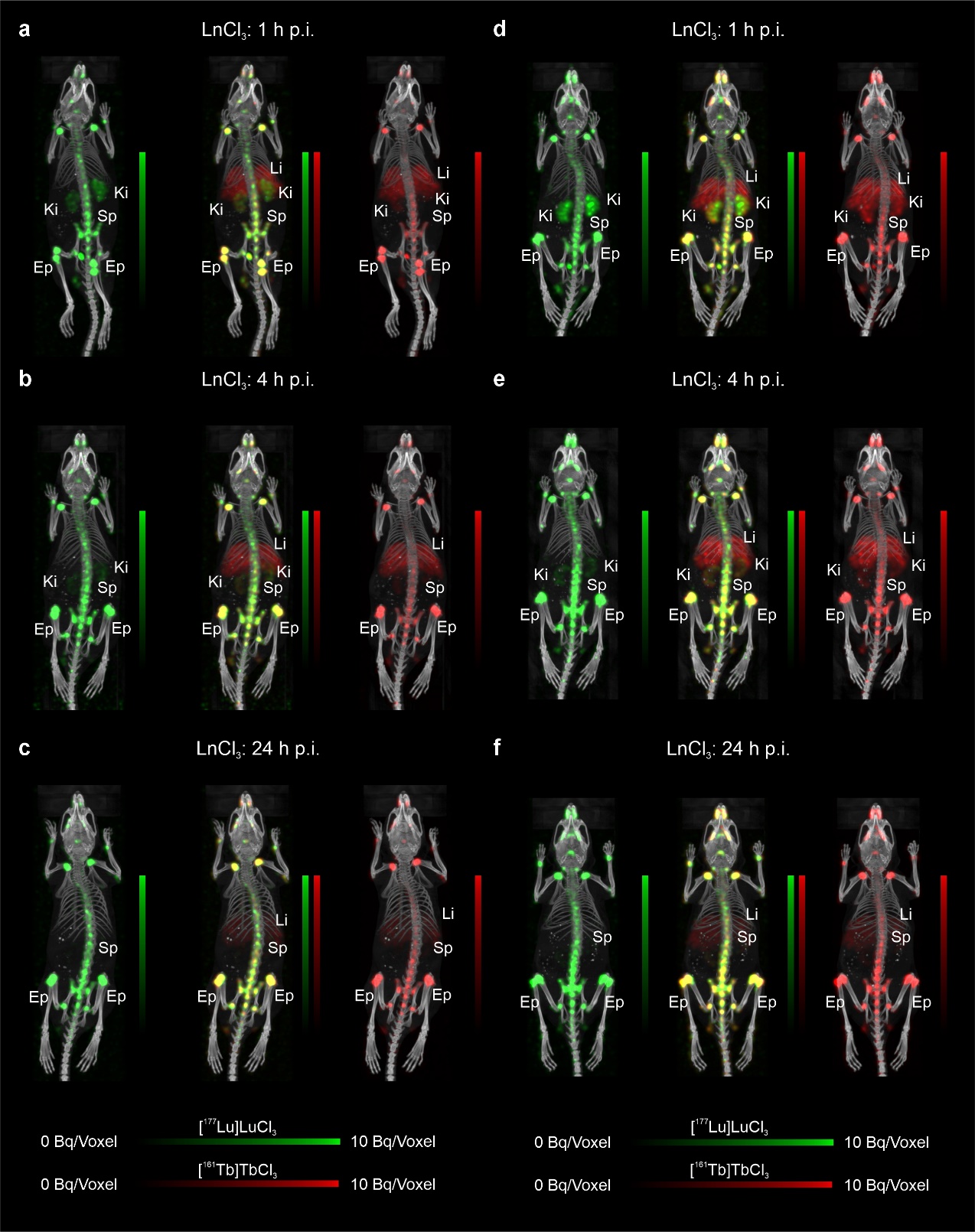


**Fig. S10** **a‒f** Dual-isotope SPECT/CT images of two representative FVB mice (**a‒c** and **d‒e**) injected with a mixture of [^177^Lu]LuCl_3_ (10 MBq, 13.8 pmol) and [^161^Tb]TbCl_3_ (10 MBq, 14.3 pmol) in acidic formulation; **a/d** SPECT/CT images at 1 h p.i.; **b/e** SPECT/CT images at 4 h p.i.; **c/f** SPECT/CT images at 24 h p.i.; (Ki, kidney; Li, liver; Sp, spine; Ep, epiphysis (indicated only for the knee region))


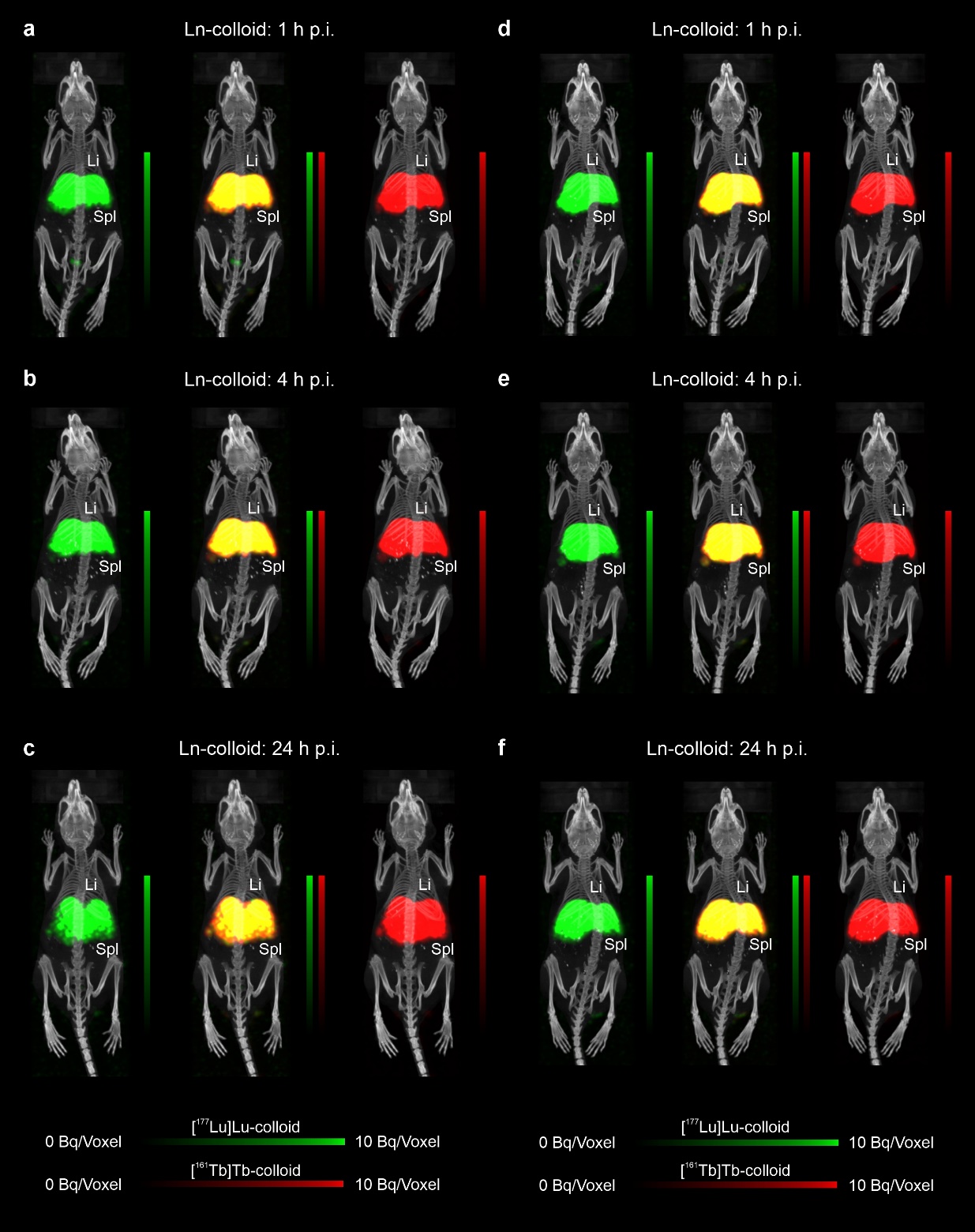


**Fig. S11** **a‒f** Dual-isotope SPECT/CT images of two representative FVB mice (**a‒c** and **d‒e**) injected with a mixture of colloidal lutetium-177 (10 MBq, 13.8 pmol) and terbium-161 (10 MBq, 14.3 pmol) in pH-neutral formulation; **a/d** SPECT/CT images at 1 h p.i.; **b/e** SPECT/CT images at 4 h p.i.; **c/f** SPECT/CT images at 24 h p.i.; (Li, liver; Spl, spleen)

**References**

1. Siwowska K, Haller S, Bortoli F, Benesova M, Groehn V, Bernhardt P, et al. Preclinical comparison of albumin-binding radiofolates: impact of linker entities on the in vitro and in vivo properties. Mol Pharm. 2017;14:523-32. doi:10.1021/acs.molpharmaceut.6b01010.

2. Deberle LM, Benešova M, Becker AE, Ratz M, Guzik P, Schibli R, et al. Novel synthetic strategies enable the efficient development of folate conjugates for cancer radiotheranostics. Bioconjug Chem. 2021;32:1617-28. doi:10.1021/acs.bioconjchem.1c00198.

3. Wallimann RH, Schindler P, Hensinger H, Tschan VJ, Busslinger SD, Kneuer R, et al. Inductively coupled plasma mass spectrometry - a valid method for the characterization of metal conjugates in view of the development of radiopharmaceuticals. Mol Pharm. 2023;20:2150-8. doi:10.1021/acs.molpharmaceut.2c01092.

4. Borgna F, Barritt P, Grundler PV, Talip Z, Cohrs S, Zeevaart JR, et al. Simultaneous visualization of ^161^Tb- and ^177^Lu-labeled somatostatin analogues using dual-isotope SPECT imaging. Pharmaceutics. 2021;13. doi:10.3390/pharmaceutics13040536.

5. Tschan VJ, Busslinger SD, Bernhardt P, Grundler PV, Zeevaart JR, Köster U, et al. Albumin-binding and conventional PSMA ligands in combination with ^161^Tb: biodistribution, dosimetry, and preclinical therapy. J Nucl Med. 2023;64:1625-31. doi:10.2967/jnumed.123.265524.
